# The twin cytokines interleukin-34 and CSF-1: masterful conductors of macrophage homeostasis

**DOI:** 10.7150/thno.50683

**Published:** 2021-01-01

**Authors:** Javier Muñoz-Garcia, Denis Cochonneau, Stéphane Télétchéa, Emilie Moranton, Didier Lanoe, Régis Brion, Frédéric Lézot, Marie-Françoise Heymann, Dominique Heymann

**Affiliations:** 1 Université de Nantes, Institut de Cancérologie de l'Ouest, Saint-Herblain, F-44805, France.; 2 SATT Ouest Valorisation, Nantes, France.; 3 UFIP, Université de Nantes, CNRS, UMR 6286, Nantes, France.; 4 Université de Nantes, INSERM, U1238, Nantes, France.; 5 Department of Oncology and Metabolism, Medical School, University of Sheffield, Sheffield, UK.

**Keywords:** IL-34, inflammation, macrophage differentiation, theranostics, tumor

## Abstract

Macrophages are specialized cells that control tissue homeostasis. They include non-resident and tissue-resident macrophage populations which are characterized by the expression of particular cell surface markers and the secretion of molecules with a wide range of biological functions. The differentiation and polarization of macrophages relies on specific growth factors and their receptors. Macrophage-colony stimulating factor (CSF-1) and interleukine-34 (IL-34), also known as “twin” cytokines, are part of this regluatory landscape. CSF-1 and IL-34 share a common receptor, the macrophage-colony stimulating factor receptor (CSF-1R), which is activated in a similar way by both factors and turns on identical signaling pathways. However, there is some discrete differential activation leading to specific activities. In this review, we disscuss recent progress in understanding of the role of the twin cytokines in macrophage differentiation, from their interaction with CSF-1R and the activation of signaling pathways, to their implication in macrophage polarization of non-resident and tissue-resident macrophages. A special focus on IL-34, its involvement in pathophsyiological contexts, and its potential as a theranostic target for macrophage therapy will be proposed.

## Introduction

In 1883, Eli Metchnikoff discovered a crucial biological process involved in cellular and tissue homeostasis: phagocytosis. This term describes the ability of some cells to engulf a variety of particles, from viruses to bacteria, fungi, dead cells and other solid materials 1. Specialized phagocytosis cells include granulocytes, dendritic cells and macrophages, and they are part of the innate immune system 2. Macrophages play a central role in maintaining general tissue homeostasis and are also active actors during inflammation, auto-immunity, infection, and cancer 3,4. Depending on the pathogenic context, macrophages can display a resolutive role or exarcerbate the disease. These particular properties of macrophages have been used to develop macrophage therapy. Theranostic, which combined imaging and therapeutic functionalities, have exploited macrophages as a potential tool from drug-delivery systems to particular tissues, or as a target for disease resolution 5,6,7.

**Figure 1:**
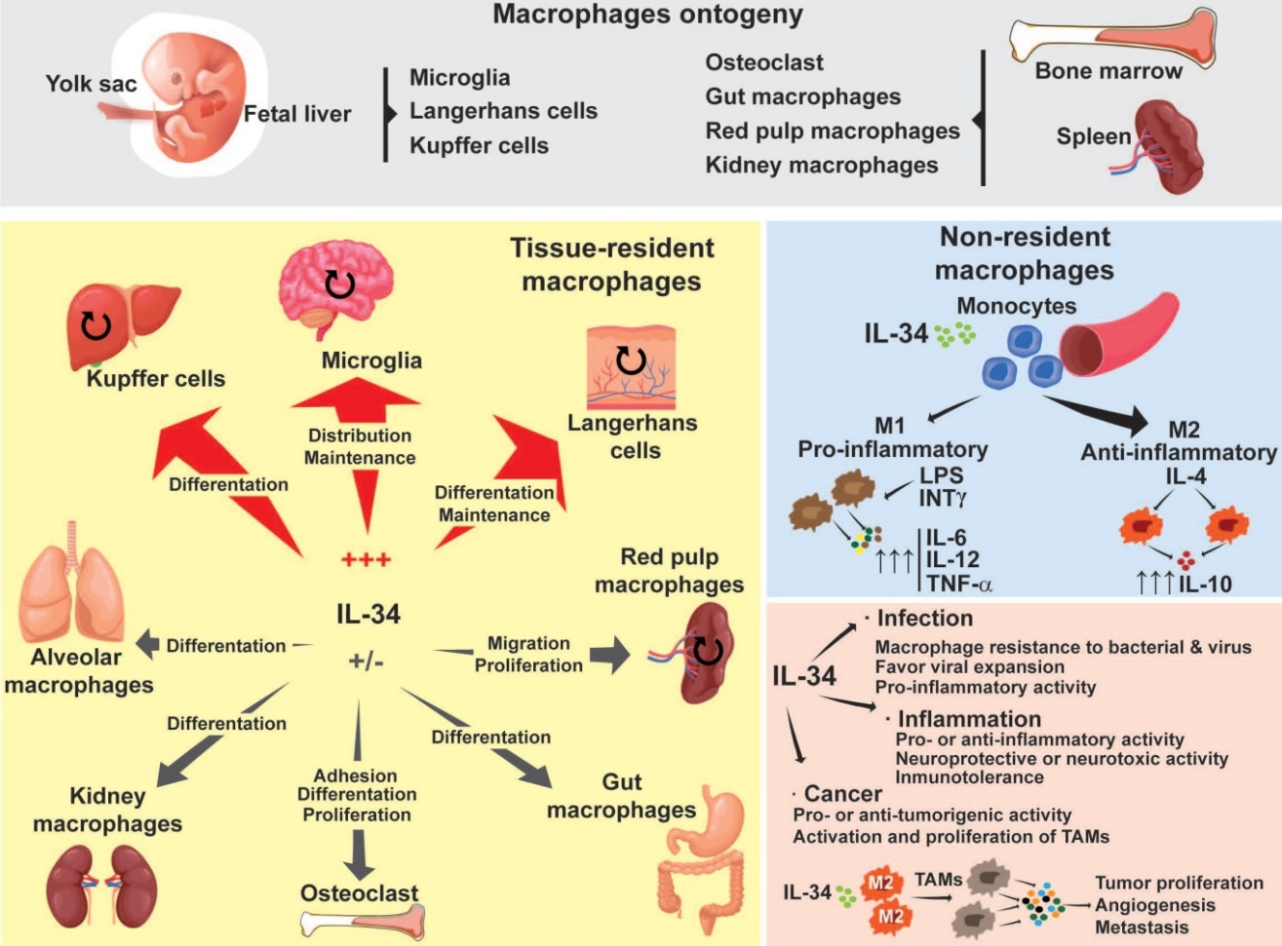
** Macrophage ontogeny and the implications of IL-34 during macrophage differentiation.** Depending on their origin, macrophages are divided into two different populations: tissue-resident macrophages and non-resident macrophages. Tissue-resident macrophages originate in the embryonic yolk sac, fetal liver, and bone marrow. Tissue-resident macrophages are capable of self-renewal of their own population (round arrows). However, in pathogenic situations, non-resident macrophages can migrate into the affected tissues and replenish the local populations by acquiring tissue specificities. Depending on the tissue, IL-34 drives macrophage differentiation, proliferation, maintenance, migration, and adhesion. Non-resident macrophages originate in the bone marrow and spleen. Circulating monocytes can extravasate and migrate to different tissues where, through the actions of different growth factors, they induce their polarization into M1 or M2 subtypes. M1 macrophages detect pathogenic particles or inflammatory molecules such as LPS or INT-γ and display pro-inflammatory functions by secreting pro-inflammatory factors such as TNF-α, IL-6 and Il-12. M2 macrophages are sensitive to molecules such as IL-4 or IL-13 and display an anti-inflammatory profile by producing soluble factors such as IL-10. IL-34 mainly induces the polarization of monocytes into an M2 subset. In pathological situations such as bacterial or viral infection, or inflammation, IL-34 can act as a pro- or anti-viral/inflammatory agent. In cancer, IL-34 behaves in a pro- or anti-tumor manner. IL-34 also induces macrophage differentiation into tumor-associated macrophages (TAMs), which are characterized by an M2 phenotype that promotes tumor proliferation, angiogenesis, and metastasis. The capacity of IL-34 to act in a positive or negative direction is tissue- and microenvironment-dependent.

Following the initial classification established by van Furth and Cohn in 1968, macrophages were considered to be part of the mononuclear phagocyte system, originating from hematopoietic stem cells located in the bone marrow 8. Although this classification is still used, studies working on specific tissue macrophages in mice over the last few years have suggested an ontogeny dichotomy in macrophages 9,10,11. According to these studies, one pool of macrophages originates in the hematopoietic stem cell lineage in the bone marrow (**Figure 1**). These macrophages, known as “non-resident” macrophages, are some of the circulating monocytes that can extravasate from blood to tissues and enrich the local population of macrophages. The other pool of macrophages, known as “tissue-resident” macrophages, originates in the yolk sac and fetal liver during embryonic development (**Figure 1**) 12,13. Tissue-resident macrophages include specialized macrophages such as the microglia in the neural system, Kupffer cells in the liver, or Langerhans cells in the skin. They are responsible for the homeostasis, development and maintenance of each specific tissue 14. In adult tissues, the local population of macrophages is maintained by autonomous proliferation and can be reinforced by macrophages migrating from the bone marrow 15. Independently of their origin, the plasticity of macrophages allows them to express conventional surface markers as well as tissue specific markers, adding an additional layer to the complexity of classifying them. As a result, their classification varies from one author to another 12,16,17.

Regardless of their origin, the proliferation and differentiation of monocytes/macrophages rely on the interaction of specific growth factors such as Macrophage Colony Stimulating Factor (M-CSF also named Colony-Stimulating Factor-1 or CSF-1), Granulocyte Colony Stimulating Factor (G-CSF), Granulocyte-Macrophage Colony Stimulating Factor (GM-CSF or CSF-2), Interleukin(IL)-6, IL-34 and their particular receptors. The absence of a ligand or receptor either compromises the proliferation of macrophage populations, or impacts their differentiation 18,19,20,21,22,23. In the present review, we will focus on the role of the IL-34 cytokine which has strongly modified our vision of macrophage biology in the last decade. The contribution of IL-34 to the proliferation, differentiation and polarization of “non-resident” and “tissue-resident” macrophages both in healthy conditions and during pathologic situations will be described and discussed, together with their therapeutic value as theranostic tools.

## Macrophage differentiation and growth factors

Circulating monocytes originate in CFU-M precursors and can extravasate from blood to tissues to become tissue macrophages. Their terminal differentiation into macrophages is regulated by specific growth factors such as CSF-1 and IL-34 24,25. However, other growth factors, such as CSF-2 and IL-6, also modulate macrophage differentiation. IL-6 is then able to induce pro-osteoclasts to shift into macrophages via differential phosphorylation of the signal transducer and activator of transcription-3 (STAT-3) 22. Simililarly, CSF-2 specifically promoted differentiation of macrophage subpopulations characterized by high induction of the antigen-specific CD8^+^ T cell type during lymphocytic choriomeningitis virus infections 26.

### CSF-1/ CSF-1R/ IL-34: more than a “ménage à trois”

For decades, CSF-1 was considered to be the main driver in the differentation of myeloid precursors toward monocytic lineages and into macrophages. Macrophage differentiation requires the interaction between CSF-1 and its unique receptor, the CSF-1 receptor (also known as CSF-1R, c-fms or CD115). This interaction triggers a cascade of signaling pathways that promote macrophage differentiation, proliferation, survival and proper functioning 27,28,29. Nevertheless, in 2002, studies using knock-out mice for CSF-1R (Csfr^-/-^) demonstrated that CSF-1R deficient mice exhibited a more severe osteopetrotic phenotype and depletion of the macrophage pool, including microglia and Langerhans cells, than CSF-1 deficient mice (Csf1^op/op^) 18,30,31,32. These observations suggested the existence of a second ligand for CSF-1R. In 2008, a screening study analyzing the interactions between secreted proteins and receptors in cell-cell signaling models identified a new cytokine that promoted monocyte survival in a CSF-1R dependent manner 25. This protein was named IL-34 and became the twin cytokine to CSF-1, sharing a common receptor. Later on, a series of publications described the essential role of the IL-34/CSF-1R interaction in the development and maintenance of osteoclast precursors, microglia and Langerhans cells 20,33,34. CSF-1R is the only hematopoietic receptor with two different ligands (CSF-1 and IL-34).

**Figure 2:**
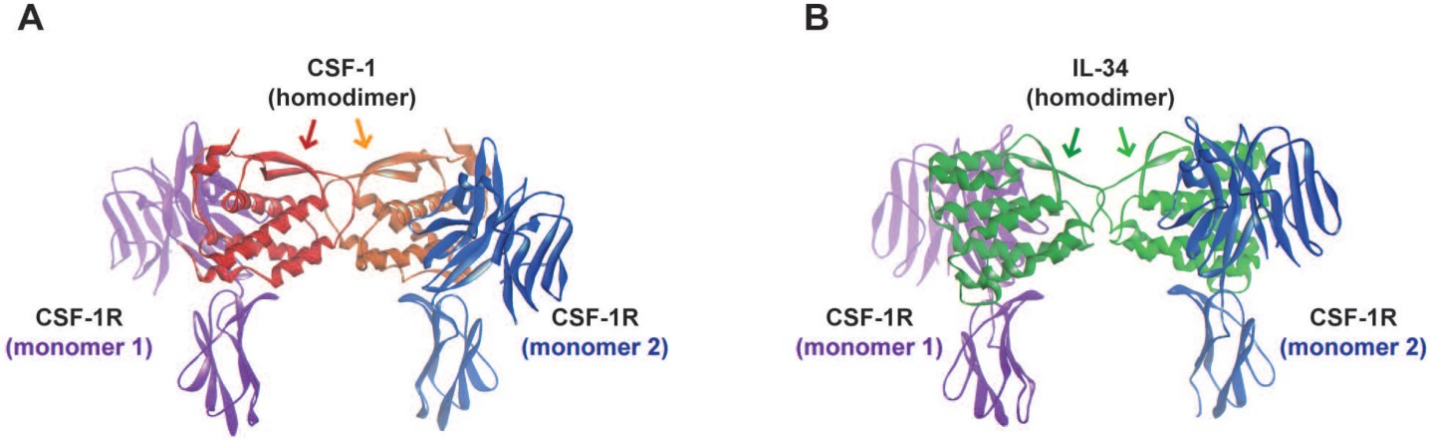
** Molecular modeling of CSF-1R binding to its ligands.** Molecular modeling was generated as described in** A**) representation of the three-dimensional crystal structure of the CSF-1/CSF-1R complex. In red and orange: monomers of CSF-1; and in blue and purple: monomers of CSF-1R. **B**) Representation of the three-dimensional crystal structure of the IL-34/CSF-1R complex. In green and light green: monomers of IL-34; and in blue and purple: monomers of CSF-1R.

**Figure 3:**
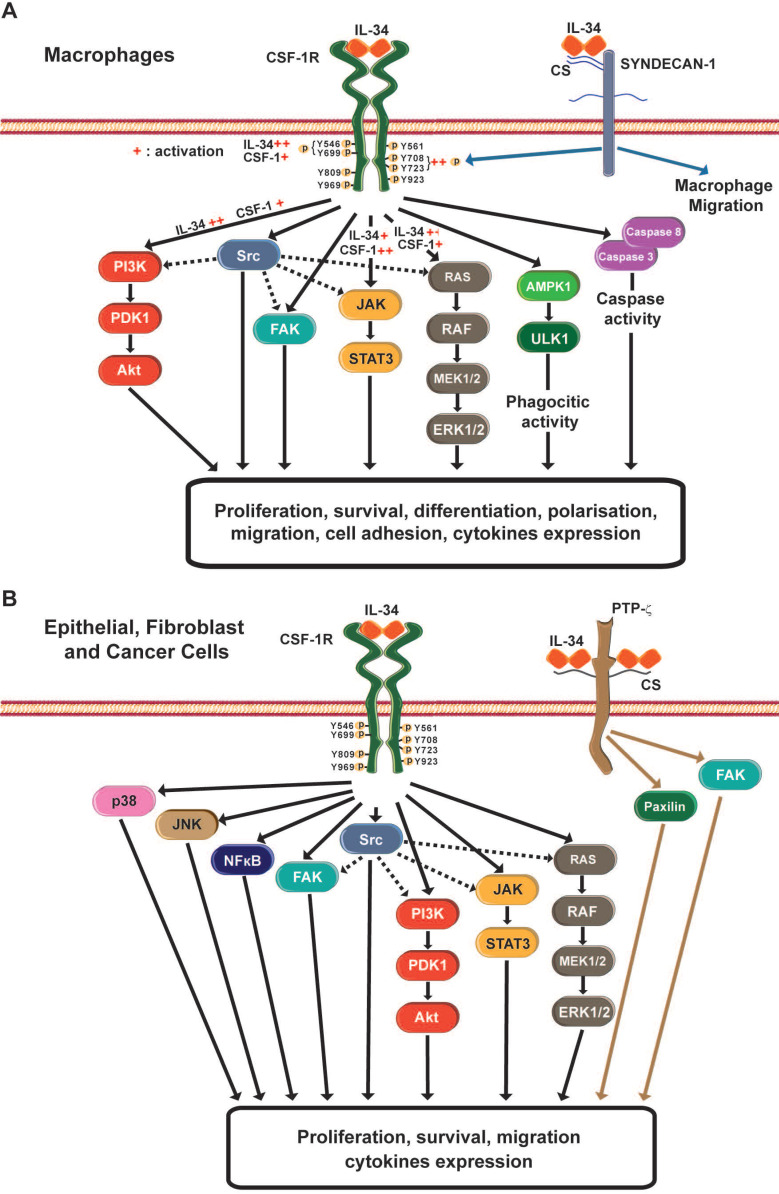
** IL-34 signaling pathways involved in macrophage differentiation and non-monocyte cells. A**) Various stimuli, such as bacterial or viral infections, pro-inflammatory cytokines, DNA damage, or chemical molecules, modulate IL-34 expression. IL-34 binds to CSF-1R or to syndecan-1 receptors expressed at the cell surface of monocytes/macrophages. The binding of IL-34 to CSF-1R induces activation of CSF-1R through auto-phosphorylation of the different tyrosines present in the cytosolic domain of CSF-1R. Compared to CSF-1, IL-34 induces strong and transient activation of CSF-1R, as well as rapid downregulation of CSF-1R. These differences between the two cytokines imply differential activation of downstream signaling pathways that result in a diversity of macrophage biological processes such as differentiation, proliferation, survival, or migration. The binding of IL-34 to the chondroitin chains of syndecan-1 results in *in vitro* phosphorylations of the tyrosines Y708 and Y723 of CSF-1R, suggesting that the complex IL-34/syndecan-1 acts as a regulator of CSF-1R activity. Moreover, IL-34/syndecan-1 interaction regulates macrophage migration. **B**) IL-34 expression in the microenvironment of epithelial cells, fibroblasts and tumor cells can induce, via CD155, activation of the different signaling pathways implicated in biological functions such as cell proliferation, migration, survival, and cytokines. IL-34 also binds to RPTP-ζ, and controls inhibition of migration and proliferation of tumor cells lines such as glioblastoma U251.

The interactions between CSF-1R and its two ligands, CSF-1 and IL-34, have been described in 29. Both ligands are present in a homodimeric form that also activates and promotes the dimerization of CSF-1R (**Figure 2**). In term of cross-species reactivity, porcine CSF-1 shared the same activity as human CSF-1, and porcine CSF-1 was able to activate mouse, cat, dog and human CSF-1R 35. However, the biological activity of human CSF-1 on mouse CSF-1R is unilateral. In the case of IL-34, human and mouse IL-34 activated porcine CSF-1R, but both cytokines presented partial cross-reactivity 35. The cytokine CSF-1 binds to CSF-1R in a hydrophilic manner, whereas IL-34 binds to CSF-1R in a hydrophobic manner 36. The nature of these molecular interactions established that each cytokine presented specific kinetics of association with CSF-1R (**Table 1**). The CSF-1/CSF-1R complex was characterized by a quick dissociation kinetic compared to the IL-34/CSF-1R complex. Futhermore, *in vitro* experiments showed a lower dissociation kinectic of the IL-34/CSF-1R complex, which may be associated with a longer span of cell signaling pathway activation and differential biological functions 24,25,36,37. The binding of IL-34 to CSF-1R results in the activation of multiple signaling pathways, including Extracellular signal Regulated protein Kinases 1 and 2 (ERK1/2), Focal Adhesion Kinase (FAK), Janus Kinase (JAK); c-JUN N-terminal Kinase (JNK), p38 mitogen-activated kinase, PhosphoInositide 3-Kinase (PI3K)/AKT, transcription factor Nuclear Factor kappa Beta (NFκB), STAT-3 and the Scr kinase family (**Figure 3**) 38,39,40,41,42,43,44,45,46,47. The CSF-1R receptor bound to its ligands by different domains, inducing differential activation of the receptor and subsequential bioactivities 38. Compared to CSF-1, IL-34 induced strong but transitory phosphorylation of CSF-1R tyrosines and downstream proteins, with rapid downregulation of the receptor 38. The differential binding properties of CSF-1 and IL-34 to CSF-1R may explain the variety and degree of activation of the different signaling pathways and their biological outcomes. Both cytokines can also work in a dual manner, showing additive and competitive biological properties 48. Moreover, CSF-1 and IL-34 were able to generate a heterodimer that may play specific roles in CSF-1R signaling, as observed for IL-12 or IL-17 49,50. These data suggest that the interaction of CSF-1R and its ligands relies on a more complex relation than the proposed “ménage à trois” and that the formation of new heterodimers between IL-34 and other cytokines should be not excluded.

### IL-34, a promiscouos cytokine with therapeutic potential

CSF-1R is not the only receptor for IL-34. Two additional receptors have been proposed: the receptor type Protein-Tyrosine Phosphatase-zeta (RPTP-ζ) and the transmembrane heparin sulfate proteoglycan syndecan-1 (or CD138) 52,53. The high expression of IL-34 in different areas of the adult brain where CSF-1R was absent suggested the existence of an additional receptor for IL-34 54. By using a CSF-1R depleted U251 glioblastoma cell line and affinity chromatography, Nandi *et al.* 52 demonstrated that IL-34 bound specifically to RPTP-ζ in a chondroitin sulfate-dependent manner. The interaction between IL-34 and RPTP-ζ induced tyrosine phosphorylations of the FAK and paxilin proteins, impairing the cell proliferation and motility of U251 glioblastoma cells. These results suggest that the IL-34/RPTP-ζ complex acts as a tumurigenic suppressor in glioblastoma 52. RPTP-ζ was also expressed in the instestinal tissue of healthy context, mainly in the colon, whereas IL-34 was mainly expressed in the ileum. However, in inflammatory bowel diseases, IL-34 was coexpressed in the same regions as RPTP-ζ and CSF-1R 55. IL-34 was over-expressed in colorectal cancer (CRC) tissues and RPTP-ζ was also expressed in tumoral and non-tumoral areas of CRC samples 56. However, an increase in CSF-1R expression alone, and not RPTP-ζ was observed in CRC cells 56. Additional studies are needed to decipher the role of the IL-34/RPTP-ζ complex in these tissues.

IL-34 can also bind to syndecan-1 in a low affinity manner 53. Segaliny *et al.* 53 showed that syndecan-1 modulated the phosphorylation of CSF-1R induced by IL-34, proposing that syndecan-1 could act as a regulator of IL-34 bioavailibility. Moreover, syndecan-1 controlled the macrophage migration induced *in vitro* by IL-34 53.

Three isoforms of CSF-1 are described: a secreted glycoprotein, a secreted proteoglycan and a membrane-spanning cell surface glycoprotein 57. Two isoforms of IL-34 generated by mRNA alternative splicing are described and recently, Ogawa *et al.* 58 showed the existence of a third isoform bound to the cell surface. In secondary lymphoid tissue, follicular dendritic cells (FDC) expressed IL-34 that induced, via CSF-1R, the differentiation of a novel class of monocytes, named FDC-induced monocytic cells. IL-34 needs the participation of the molecular chaperone 78-KDa glucose-regulated protein (GRP78) 58. How the cell-surface IL-34 variant induces the differentiation of monocytes remains unclear. All these data are evidence of new potential targets that need to be taken into account when developing further therapies against IL-34.

## From monocytes to “non-resident” macrophages

### Regulation of M1 and M2 differentiation by IL-34

**Figure 4:**
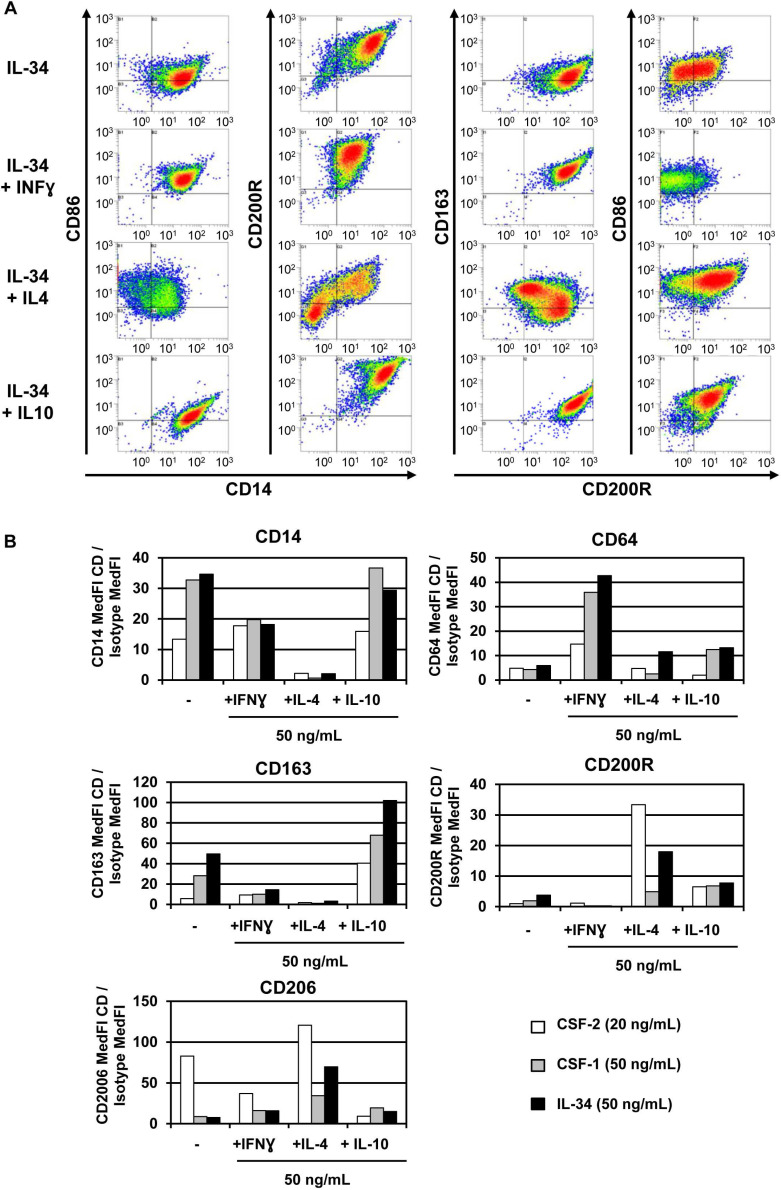
** IL-34 is a pro-M2 macrophage differentiation factor.** Macrophage isolation and treatment were performed as described in **A**) IL-34 treatment induced macrophage differentiation with an M2 phenotype, alone or in combination with IL-4 and IL-10. Macrophages were treated with IL-34 (50 ng/ml) or in combination with IFN-γ (50 ng/ml; pro M1), IL-4 (50 ng/ml; pro M2a), and IL-10 (50 ng/ml; pro M2c), for 2 days and cells were analyzed by means of flow cytometry using specific antibodies for both M1-like macrophages (CD14, CD86 and CD64) and M2-like macrophages (CD163, CD200R and CD206). **B**) Comparison of macrophage differentiation after treatment with the cytokines CSF-2 (20 ng/ml), CSF-1 (50 ng/ml) and IL-34 (50 ng/ml) alone or in combination with IFN-γ, IL-4 or IL-10 as performed in A. The three cytokines in combination with IFN-γ were able to induce M1 macrophage differentiation as shown by the increase in CD64, an M1 marker. IL-34 modulates M2 markers (CD163, CD200R and CD206) alone or in combination with IL-4 and IL-10. No effect of IL-34 was observed in CD14 expression. Overall, IL-34 was able to induce M1 and M2 macrophage differentiation with a specific increase in CD163, an M2 marker.

In healthy conditions, CSF-1 and IL-34 act identically to promote macrophage differentiation and survival via their common receptor, CSF-1R. In human monocytes, CSF-1 and IL-34 activate similar signaling pathways (STAT, AKT, ERK1/2) that trigger the proliferation of circulating monocytes and their differentiation into macrophages 59. Moreover, both cytokines induce autophagy by activating AMPK and ULK1 pathways and caspase-3 and-8 activities, two essential properties of macrophages 59. Depending on their microenvironment, naive-circulating monocytes differentiate into two subtypes of macrophage: M1 and M2. M1 “pro-inflammatory” macrophages respond to pro-inflammatory molecules, such as interferon gamma (IFN-γ) and lipopolysaccharides (LPS), by up-regulating IL-6, IL-12 and TNF-α and promoting activation of the immune response via Th1. M2 “anti-inflammatory” macrophages respond to IL-4 stimulation by up-regulating IL-10 and promoting activation of Th2 60,61,62. Treating circulating monocytes with IL-34 induced macrophage differentiation into the M2 type (CD14^+^ CD163^+^), with high production of IL-10 and low expression of IL-12 (**Figure 4**). This differentiation could be reversed to the M1 type by CSF-2 and INF-γ treatments 63. In agreement with these results, Lindau *et al.* 64 showed that IL-34 expressed at the fetal-maternal interface also induced polarization of macrophages into CD14^+^ CD163^+^ with production of IL-10 that contributes to a local immune-tolerant environment. In addition, CSF-1- and IL-34-activated macrophages show various polarization potential of macrophages in the immune response 59. CSF-1-differentiated M1 macrophages enhanced naive T lymphocyte polarization into Th1 better than IL-34-differentiated M1 macrophages. However, no differences between either cytokine-differentiated macrophages were observed with respect to Th2 cell polarization 59. Moreover, using a human leukemia model, IL-34 enhanced the differentiation of leukemia cells into differentiated macrophages by means of an increase in CD14 or CD68 and a decrease in CD71, a cell surface marker for immature myeloid cells. This suggests that IL-34 is capable of reprogramming leukemia cells from a naive state to mature and functional monocytes 65.

This dual polarization in macrophage differentiation between CSF-1 and IL-34 is also observed in species other than humans and rodents. In birds, chicken IL-34 interacted with CSF-1R, triggering the activation of multiple signaling pathways (JAK, STAT 1/3, NFκB, TYK2, MAPK) and the induction of a specific pro-inflammatory response by up-regulating the secretion of Th1 and Th17 cytokines 47. In frogs, IL-34 and not CSF1-differentiated macrophages showed an ability to resist bacterial infections 66. In fish, rainbow trout IL-34 was expressed with relatively high levels along the tissues compared to CSF-1, which showed variable expression levels and was presented in this fish 67. Moreover, IL-34 expression, but not CSF-1, increased significantly during the inflammatory process and induced macrophage proliferation 67. In grass carp, IL-34 showed a similar capacity with regard to macrophage differentiation in an inflammatory context 68. In the mudskipper fish, IL-34 levels increased after bacterial infection and induced macrophage differentiation with high phagocytic activity in a CSF1R-dependent manner 69. In Japanese flounder, IL-34 induced an inflammatory response against bacterial infection characterized by production of pro-inflammatory cytokines, ROS, acid phosphatase activity and inducing cellular resistance. Moreover, overexpression of IL-34 showed tissue-dependent expression of pro- and anti-inflammatory mediators via JAK/STAT signaling pathways, depending on the manner that was associated with infection resolution 70. In this context, IL-34 could be used as an adjuvant to DNA vaccine treatment against nocardiosis infection in fish 71. Finally, zebrafish have been suggested as a good model for studying the role of IL-34 during brain development or in brain disorders and liver or skin diseases 72,73,74.

*In vitro* data show that IL-34 favors the differentiation of circulating monocytes to the M2 subtype instead of M1, whereas CSF-1 mainly promotes M1 differentiation. These results suggest that in healthy conditions, each cytokine participates in the expansion of the various subtypes of monocytes/tissue macrophages to contribute to the tissue's immune homeostasis. However, in pathogenic situations or tissue damage, and depending on the microenvironment, both cytokines can act as pro-inflammatory agents, as was observed during pathogen infections in different species. The role of IL-34 to promote macrophage differentiation in a pro-inflammatory or anti-inflammatory subtype must be conditioned to complex signaling activation and its particularity according to the species, tissue, and microenvironment context.

### IL-34-differentiated macrophages in viral infections

During viral infection, macrophages play an essential role, detecting virus particles and triggering an anti-viral immune response through the production of a large variety of cytokines.

Infection by the human immunodeficiency virus-1 (HIV-1) is characterized by the loss of T lymphocytes in a progressive manner and susceptibility to opportunistic infections 75. IL-34-induced macrophages were characterized by better resistance to HIV-1 infection than CSF1-differentiated cells 76. The HIV-1 resistance of IL-34-macrophages lay in the specific expression of restriction factor genes APOBEC, IFITM and SAMHD, blocking the replication progress of the virus 76. However, while the immune system slowed down the progression of HIV-1, the virus invaded the central nervous system (CNS) in the early stages of infection, inducing severe neurotoxic effects 77. Mathews *et al.* 78 engineered a humanized mouse model that produces human microglia and mimics viral infections. The authors demonstrated that IL-34 was responsible for human microglia proliferation in the mouse's brain. The IL-34 microglia favored HIV-1 infection *in vitro*, induced inflammation and a neurotoxic response, and formed a large reservoir for virus particles 78. These results suggest that Il-34 functions depend on the monocyte linage, tissue, and microenvironment context.

Influenza viruses are characterized by the production of seasonal epidemic disease that can occasionally generate global pandemics 79. Patients infected with influenza A virus (IAV) secreted high levels of IL-34 in blood serum. In addition, IAV infection stimulated the production of IL-22, which displays a protective role on lung epithelium during IAV infection 80. Interestingly, IL-22 induced the expression of IL-34, and the accumulation of IL-34 suppressed IL-22 production in a negative feedback loop. These results suggest that IL-34 promoted the activation of an inflammatory response in influenza virus infection by suppressing the positive effect of IL-22 80.

Infection by hepatitis C virus (HCV) is associated with the formation of chronic liver diseases such as liver fibrosis, and IL-34 may contribute to this pathogenesis. Patients with HCV presented high levels of CSF-1 and IL-34 in their blood serum 81. HCV infection induced the production of both cytokines by hepatocyte cells, and this increased macrophage proliferation and differentiation with profibrogenic properties. In turn, macrophages negatively regulated NK cells, promoting the survival and activation of stellate cells, which secreted type I collagen. Concomitantly, the production of IL-13 during liver fibrosis enhanced the synthesis of type I collagen by decreasing expression of the collagenase MMP-1 81. As with HCV, hepatitis B virus (HBV) infections can lead to hepatic fibrosis and chronic inflammation in which IL-34 may be involved. In a rat pre-clinical model, IL-34 inhibited the replication of HBV, and HBV patients presented significantly lower levels of IL-34 in their blood serum compared to healthy donors 82. Interestingly, the level of IL-34 detected in blood plasma differed according to the phases of chronic HBV infection and correlated with progression of liver fibrosis and poor prognosis 83. The discrepancy between the two studies can be explained by the chronicity of the infected patients, or by the accuracy of the different methods selected for IL-34 quantification in serum. As observed in HCV infections 81, IL-34 levels correlated with the chronicity of the HBV infection. However further studies are needed to clarify the functional relationship between IL-34 and HBV infection. HBV is also a major factor associated with the development of hepatocellular carcinoma (HCC). Expression of the HBx viral particle in HCC-infected cells induced expression of IL-34, which promoted cancer cell proliferation and migration via CSF-1R and syndecan-1 receptors in an ERK- and STAT3-dependent manner 84.

Infection by the Hantaan virus causes Hemorrhagic Fever Renal Syndrome (HFRS). Patients with HFRS show high levels of plasma IL-34, correlating with an increase in phagocytic (CD14^+^CD16^-^) and inflammatory (CD14^+^CD16^+^) monocytes. IL-34 may then contribute to the virus' expansion 85.

IL-34 plays an important role during viral infections in other species. In the grass carp, infection by grass carp reovirus II induced the expression of IL-34 and generated a pro-inflammatory response by producing IL-1β, IL-6 and IL-8, and inhibiting anti-inflammatory factors such as IL-10 and the transforming growth factor β1 (TGF-β1) 68. Amphibian populations are dramatically affected by the frog virus 3 (FV3) ranavirus 86. In *Xenopus laevis* CSF-1 and IL-34 polarized macrophage differentiation with distinct functionalities 86. Both subtypes of macrophage expressed specific pattern recognition receptors (PRRs) that were essential for pathogen recognition. IL-34-activated macrophages highly expressed antiviral interferon genes, showing better anti-FV3 properties than CSF1-activated macrophages. Similar to HIV-1 virus infection, FV3 infection induced the expression of the antiviral restriction factors IFNX, INOS and APOBEC in IL-34-activated macrophages 87. Consequently, an increase in toll-like receptor 2 and 4 transcripts was detected in macrophages implicated in the recognition of bacterial cell wall LPS, as well as in the secretion of antiviral interferon IFN7 and tumor necrosis factor-alpha (TNF-α).

Overall, these data suggest that IL-34 induces differential transcription programs and functions compared to CSF-1 during viral infection in high vertebrates and that IL-34 is able to display antagonist roles during viral infection. IL-34 can firstly induce antiviral response by activation of a specific set of genes that block viral replication, as has been observed in HIV-1 and FV3 viral infection. Secondly, hyper activation of pro-inflammatory by IL-34 in response to viral infection promotes viral expansion and tissue damage as has been observed in HCV, HBV, and HFRS affected patients. In both cases, IL-34 could be considered as an interesting candidate for generation of antiviral treatments or as a target to reduce its pathogenic contribution during viral infection.

### Effects of IL-34 in tumor-associated macrophage polarization

One characteristic of tumorigenesis is the infiltration of macrophages into the affected tissue or organs. These macrophages are known as tumor-associated macrophages (TAMs). TAMs are considered to be heterogenous populations that originate in adult circulating myeloid precursors and “tissue-resident” macrophage precursors 62. TAMs are responsible for driving pro- or anti-inflammatory responses by controlling immunocompetent, stroma and vascular cells according to the tumor microenvironment (TME), 89. TAMs can be classified into two subtypes: M2 macrophages, which favor tumor progression, angiogenesis and metastasis, and M1 macrophages, which facilitate local inflammation leading to the anti-tumor response 90. However, depending on the surrounding TME, TAMs acquire M1, M2 or dual polarization 91. Recent advances in TAM functions and their involvement in cancer and inflammatory diseases have been reviewed in 91,92,93.

TAM differentiation and polarization are driven differently by CSF-1 and IL-34 94. Several publications have demonstrated the role of IL-34 in TAM polarization and the pro-tumorigenic effect of IL-34-derived TAMs. For instance, IL-34 facilitated macrophage extravasation and polarization toward the M2 phenotype and promoted osteosarcoma proliferation and metastasis expansion 95. In lung cancer, tumor cells express IL-34, which induced TAM polarization into an M2 pro-tumorigenic phenotype with the properties of chemoresistance to tumor cells through Akt signaling pathway activation 96. Another study showed that nitric oxide therapy reduced tumor progression and correlated with a decrease in the IL-34-derived TAM populations in tumorigenic castration-resistant prostate cancer xenografts 97. Taken together, these observations suggest that IL-34 may play an essential role in both the pro-tumorigenic polarization of TAMs, and tumor progression, as revealed in various other cancers. In colorectal cancer, high levels of IL-34 correlated with TAM infiltration and a poor prognosis for the affected patients 98. In ovarian cancer, cytotoxic chemotherapy enhanced the expression of IL-34, CSF1R and associated TAMs, correlating with poor patient survival 99. Similar observations were obtained in hepatocellular carcinoma 100,101. However, the results were more contrasted in breast cancer in which IL-34 displayed different prognosis properties depending on the cancer subtype. High levels of IL-34 were correlated with better survival and good prognosis in the luminal B and HER2 subtypes, whereas IL-34 was associated with poor prognosis in basal breast tumors 46. The different behavior of IL-34 in breast cancer prognosis could be explained by the differential expression of IL-34 in basal cells between the luminal B and HER2 subtypes. IL-34 expression promoted migration of the basal cell subtype (independently of CSF-1R), but no major effect was observed in the luminal and HER2 subtypes, where cell migration was associated with CSF-1 expression. Cell signaling activation by IL-34 also varied depending on subcellular subtype and its interaction with other receptors, suggesting that IL-34 has a differential role depending on the breast tumor subtype. In relation to immune cells, CSF-1 was also directly related to pro-tumorigenic macrophage M2 differentiation, but not IL-34, which was associated with M1 macrophage differentiation in the luminal B subtype. In the same way, immune cell infiltration in tumor regions was associated with CSF-1 and not IL-34. All these data suggest that IL-34 can act as an anti-tumorigenic factor in the luminal B and HER2 breast tumor subtypes, by promoting M1 macrophage differentiation, and as a pro-tumorigenic factor in basal subtypes, by promoting tumor cell migration.

IL-34 can be considered to be a key cytokine that induces specific TAM polarization with pro-tumogineric activity. With the exception of certain subtypes of breast cancer, an increase in IL-34 in the serum has been associated with poor prognosis. This indicates that IL-34 might be considered as a potential taget for developing new treatments for reducing TAM and tumor proliferation.

## Tissue-resident macrophages and IL-34

In healthy tissues, the local population of macrophages is sustained by autonomous proliferation 15. During pathologic episodes, the death of local macrophages induces the infiltration of circulating monocytes to reinforce the local population until proliferation of the remaining resident macrophages. These circulating monocytes end up acquiring tissue-resident macrophage features, indicating the presence of local signals that confer tissue-specific identities 102. Several authors have suggested that the autonomous proliferation of local macrophages is regulated by the existence of a tissue “niche”. Each tissue niche consists in cell-cell circuits based on the mutual benefits of stroma cells and tissue-resident immune cells by means of the secretion of specific factors []. The following sections will be dedicated to the role of IL-34 in the differentiation of tissue-resident macrophages.

### IL-34 in bone and auto-immune diseases

Alterations in bone homeostasis can be translated into an imbalance between osteoblast and osteoclast populations, which leads to bone malformations and diseases such as osteopetrosis or osteoporosis. As part of the tissue-specific mononuclear phagocyte lineage, osteoclasts play an essential role in maintaining bone homeostasis. A key factor in the differentiation of osteoclast progenitors is the Receptor Activator of Nuclear Factor kappa-B ligand (RANKL)^104^,^105^. In addition, other factors, such as IL-6 and CSF-1, contribute to the differentiation of osteoclasts in collaboration with RANKL, which is mandatory for osteoclastogenesis 106,107,108. As already mentioned, in studies using knockout mice for CSF-1 and CSF-1R, IL-34 circumvented the observed osteopetrosis phenotype, suggesting that IL-34 may play a role in osteoclast generation. In 2010, Baud'Huin *et al.* 40 demonstrated that a combined treatment with IL-34 and RANKL induced osteoclastogenesis in humans and mice, and that IL-34 can replace CSF-1 in osteoclastogenesis. RANKL is thus mandatory in osteoclastogeneis, in contrast to IL-34 which can be replaced by other soluble factors. Both cytokines, CSF-1 and IL-34, were able to regulate the adhesion, differentiation and proliferation of osteoclast precursors but not osteoclast survival in the bone marrow. Furthermore, human and mouse IL-34/RANKL differentiated osteoclasts presented bone reabsorbing activities 109. In the bone marrow, TNF-α stimulates the expression of IL-34 and CSF-1 in osteoblasts via the NFκB pathway. In pathologic situations such as osteopetrosis, the spleen acts as a reservoir for osteoclast progenitors. In a vitamin D-dependent manner, splenic vascular endothelial cells expressed IL-34, which was needed to maintain and conscribe osteoclast progenitors from the spleen to the bone tissue. Recently, a study using bone marrow macrophages from mice reported the promoting function of IL-34 and RANKL in osteoclast differentiation via activation of the JAK2/STAT3 signaling pathway. This activation was reversed in the presence of the protease inhibitor AG490, which also favored the expression of SMAD7, an antagonist TGF-β protein for the STAT pathway. In an oncological context, osteosarcoma cells expressed IL-34 and this expression was regulated by TNF-α and IL1-β 95. IL-34 then appeared as a pro-proliferative and metastatic factor in osteosarcoma, with a potential role in angiogenesis via glycosaminoglycan. In relation with this pro-angiogenic effect, IL-34 promoted macrophage extravasation and polarization to an M2 phenotype 95.

Additionally, IL-34 plays a regulatory role in inflammatory diseases. In the past few years, an increasing number of publications have associated IL-34 with a bad prognosis in rheumatoid arthritis (RA) 111,112,113,114. Chemel *et al.* 111 was the first to describe the correlation between IL-34 and inflammation levels in RA and found that IL-34 expression was upregulated by IL-1β and TNF-α stimulation in synoviocytes. In contrast, two members of the transforming growth factor β family, BMP-2 and TGF-β1, were described as inhibiting expression of IL-34, acting as regulators of inflammation during RA. While macrophages are potential targets of IL-34, fibroblast-like synoviocytes were identified as new targets expressing CSF-1R. [116]To our knowledge, no data report the expression of RPTP-ζ by synoviocytes. The binding of IL-34 to its receptor activated the expression and secretion of IL-6 (via the JNK/P38/NF-κB signaling pathway), which in turn induced polarization of naive T lymphocytes into a Th17 population. Moreover, IL-34 activated/inhibited the expression of RANKL/OPG by fibroblast-like synoviocytes and circulating monocytes promoting cartilage and bone destruction in an IL-17-dependent manner, which was consistent with the correlation observed between increased IL-34 and RANKL levels in patients with RA 117,118. IL-34 is suspected of playing a role in various other autoimmune diseases (**Table 2**). Systemic sclerosis (SSc) affects the connective tissue of the skin, lung, bowels, and other internal organs. IL-34 levels were increased in the serum of patients with SSc and correlated with poor prognosis. Moreover, the increase in IL-34 also correlated with the development of interstitial lung disease (ILD) 146. As SSc patients also presented an increase in Th17 cells, IL-34 may enhance the proliferation of Th17 cells, contributing to the development of ILD. Systemic lupus erythematosus (SLE) is characterized by an acute nephritis process. Wada *et al.* 143 demonstrated that IL-34 and its two receptors, CSF-1R and RPTP-ζ, were highly expressed in patients with lupus nephritis. IL-34 induced circulating monocytes and intra-renal macrophage proliferation and accumulation, together with B and T lymphocyte enrichment in kidney areas, promoting the inflammation process and tubular epithelial cell apoptosis. In Sjogren's syndrome, Ciccia *et al.* 139 showed that IL-34 expression increased in the ductal epithelial cells of inflamed salivary glands. The increase in IL-34 correlated with the expansion of pro-inflammatory monocytes and expression of IL-23 and IL-17, suggesting that IL-34 plays an important role in the inflammatory process in Sjogren's syndrome.

Periodontitis is another type of bone-degenerative illness associated with osteoclastogenesis. A few studies have shown that TNF-α and IL1-β upregulated IL-34 expression by human gingival fibroblasts, which promoted osteoclastogenesis in combination with RANKL. Clinically, a positive correlation between levels of IL-34 in gingival crevicular fluids and the aggressiveness of periodontitis was observed.

In relation with myeloid differentiation, multiple myeloma (MM) is a hematological disease that affects the axial skeleton of affected patients in terms of bone fragility. MM murine cells expressed IL-34 *in vitro* which was upmodulated by inflammatory factors such as IL1-β, IL-6, TNF-α and surprisingly TGF-β. IL-34 was also detected in bone marrow fluids from MM patients. In the pathological context of MM, IL-34 promoted CD14^+^ monocyte differentiation into osteoclasts by upregulating the expression levels of osteoclastogenesis-related genes such us DC-STAMP and OC-STAPM. IL-34 did not appear essential for osteoclast differentiation in MM, but IL-34 activation accelerated the osteolytic process and bone lesions in patients^151^.

The contribution of IL-34 to osteoclastogenesis, bone-associated inflammatory diseases, and osteosarcoma cell proliferation suggests that IL-34 acts as a pathogenic molecule in bone disease. The presence of IL-34 at high concentrations in serum in the majority of patients affected by bone-associated diseases supports the idea that IL-34 must be considered to be an important target for developing new therapies that block or inhibit its activity.

### IL-34 in microglia differentiaton and neural disorders

Microglia are the resident macrophage population of the CNS and present neurotoxic and neuroprotective activities^152^. Microglia development depends on CSF-1R activity and, by consequence, its ligands CSF-1 and IL-34 18. IL-34 is predominantly expressed by neurons, whereas CSF-1 is expressed by astrocytes, microglia and oligodendrocytes. CSF-1 or IL-34 knockout mice highlighted the harmful impact of one of these two cytokines on the survival/differentiation of microglia populations in different regions of the CNS. IL-34 was not essential for the development of embryonic microglia cells but appeared crucial for their maintenance during adulthood. In agreement with this observation, Easley-Neal *et al*. 155 demonstrated the essential role played by IL-34 in microglia support during postnatal life using specific function-blocking antibodies against each CSF-1R ligands. Only CSF-1 seemed to be required to establish microglia in the embryonic brain. In adult mice, IL-34 was necessary for the development of gray matter microglia, and CSF-1 for white matter microglia. The regional localization of each cytokine correlated with the affected regions. Interestingly, regions of the brain with a mix of gray and white matters, such as the cerebellum and the dentate gyrus, showed differential responses to anti-CSF1 antibodies with no effect in the denatate gyrus and partial depletion of the microglia in the cerebelum, whereas no effect was observed for anti-IL-34 antibodies. However, treatment with both antibodies showed depletion of the microglia in both areas, suggesting the presence of a compensation mechanism between the two cytokines^155^. However, by using a zebrafish model, two independent research teams recently demonstrated that IL-34 was required for proper colonization of microglia progenitors from the yolk sac to the head region in the early stages of embryo development. Afterwards, IL-34/CSF-1R signaling and neural apoptosis determined microglia development in the different regions of the CNS 72. Additionally, IL-34 was also responsible for the distribution of tissue-resident macrophages from the yolk sac to other parts of the embryo, such as the epidermis 74. In the retina, IL-34 was mainly expressed by the retinal ganglion cells and was responsible for maintaining the retinal microglia population localized at the inner plexiform layer of the neural parenchyma. This specific microglia subtype is implicated in the feedback regulation of cone bipolar cell axons.

The peripheral nevous system (PNS) is the motor and sensitive neural network that links the CNS and the rest of the body. Although the PNS is characterized by a self-regeneration capacity, nerve injury and degeneration can occur. As with the CNS, the PNS has a macrophage resident population^158^. Recently, Wang *et al.* 159 showed that PNS macrophages originate in the embrionic yolk sac and hematopoeitic sources. Transcriptomal characterization of PNS macrophages suggested the existence of two populations associated with axons and neural cell bodies. The PNS displayed similar transcriptomic gene expression to activated microglia. Moreover, the PNS macrophage population was significantly reduced in IL-34 deficient mice, suggesting that IL-34 plays a significant role in PNS macrophage development, as observed for microglia.

As observed in other tissues, IL-34 plays an important role in pathogenic situations. Depending on the disease, IL-34 is considered either a neuroprotective or neurotoxic agent. In neurodegenerative diseases, IL-34 primarily produced by neurons promoted microglia differentiation and proliferation via the CSF-1R receptor in an Alzheimer disease (AD) mouse model. AD was characterized by the production and accumulation of neurotoxicoligomeric β-amyloid peptides. IL-34-differentiated microglia were able to abolish the neurotoxic effects of β-amyloid peptides and derivates by the production of the insulin degrading enzyme (IDE), heme oxygenase-1 (HO-1) and TGF-β. These results suggest that IL-34 may act as a neuroprotector agent and that the mechanism of action of IL-34-differentiated microglia may differ from that of CSF1-differentiated cells 154,161. A recent report revealed that IL-34 and CSF-1 cytokines induced the differentiation of microglia into a CD11c^+^ type 126. The CD11c^+^ microglia is characterized by the expression of insulin-like growth factor-1, which is important for neural myelination and survival 162. Both cytokines induced pro-inflammatory rather than anti-inflammatory activation of microglia, with a major induction of the IL-1β factor 163. These discrepancies can be partially explained by the fact that the neuroprotective role of IL-34 in AD is based in rodent models whereas the pro-inflammatory role is based in human microglia obtainedt from postmortem patients affected by AD. The function of IL-34 microglia in this pathological context can differ between the two species, and further studies will be needed to clarify the role of IL-34 in AD. Another example of IL-34 neuroprotection occurs in retinal diseases such as photoreceptor degeneration disease. Specific IL-34 retinal microglia populations massively migrate to the subretinal space during photoretinal damage to cooperate in the protection of the retinal pigment epithelium 156. On the other hand, IL-34 may display some neurotoxic effects in Huntington's disease, characterized by high expression of the amyloidogenic fragment of the Huntington protein (mHTTx1). This accumulation, which was mediated by Neural IκB Kinase (IKK)/NKKbeta, induced high expression of IL-34 by neurons, activating the local microglia and inducing the production of neurotoxic pro-inflammatory molecules 164.

Multiple sclerosis (MS) is characterized by strong demyelination of the CNS, as well as low levels of vitamin D 165 (**Table 2**). Vitamin D induced moderate expression of IL-34 by neurons that led to microglia differentiation with partial anti-inflammatory activity 128. However, *in vitro* stimulation of microglia cells with IL-34 reduced the secretion of inflammatory mediators and promoted the expression of anti-inflammatory mediators, suggesting a potential role for IL-34 in the prevention of neuron demyelination.

Several articles support the evidence that IL-34-derived microglia are necessary for viral and prion protection in the CNS. As previously mentioned, HIV-1 invades the CNS, generating severe neurotoxic effects 77. In humanized mouse models, IL-34-differentiated microglia acted as a reservoir for virus particles, promoting the persistence of HIV-1 infection 78. West Nile virus (WNV) affects neuronal synapses within the hippocampus region. IL-34-deficient mice, which are characterized by the absence or high reduction of microglia, presented normal presynaptic terminals in cases of WNV infection. These data suggest that microglia are involved in the loss of neural synapses during viral infection. However, the main role of IL-34 in this infection remains unknown 166.

Prion infections are another type of infection that generates neural degenerative diseases. Microglia play an essential role as neuroprotective agents in prion infections and their disruption results in an increase in prion malignancy 167. IL-34 secreted by neurons sustained microglia proliferation and consequently played a crucial role in the CNS 20. Conditional mice for IL-34 experienced an acceleration of prion infection compared to healthy mice, suggesting that IL-34 attenuates prion disease by inducing the expansion of microglia populations, which play a neuroprotective role in prion pathogenesis 167.

IL-34 and CSF-1 are essential cytokines for the proper development and maintenance of microglia with particular activities at different regions in the CNS during adulthood. Impairment or overproduction of both cytokines are associated with neural pathogenic situations. Depending on the disease and microenvironment, Il-34 can act as a neuroprotective or neurotoxic agent. IL-34 favors microglia differentiation and proliferation, myelination, and viral/prion protection, suggesting a potential use of IL-34 as a therapy in certain neural diseases. However, it has been also reported that in certain neural disorders, e.g. Huntington disease, IL-34 can induce microglia differentiation with neurotoxic pro-inflammatory properties and production of pro-inflammatory molecules. In this type of pathological context, a potential blocking of IL-34 activity may be lead to a promising therapeutic approach.

### Role of IL-34 in the differentiation of Langerhans cells and melanomas

Langerhans cells (LCs), together with microglia, belong to the most specialized tissue-resident macrophages. LCs are the first immune barrier of the skin and contribute to its homeostasis. As with microglia, LCs autonomously maintain their own population with minimal contribution from circulating monocytes from the bone marrow and spleen 30. Like all monocytic cells, LCs express CSF-1R, which is essential for their development, proliferation and survival 33,30. CSF-1 or IL-34 knockout mice revealed a differential functional impact for both cyctokines on LCs. The absence of IL-34, but not CSF-1, compromised the presence of the LC population in the skin 20,30. During development, LC precursors derived mainly from the fetal liver, with a reduced population originating from the yolk sac 168. In contrast to microglia, IL-34 was essential for LC development in the skin rudiment during embriogenesis in mice, and for cell maintenance in adult mice 33,169. During skin maturation, keratinocytes were the main producers of IL-34, which enhanced LC differentiation and proliferation in the skin dermis 169. CSF-1 and IL-34 exhibited differential activities in LC renewal depending on the pathophysiological context. Neutrophil-derived CSF-1 drove the renewal of LCs in the course of skin injury, while IL-34-derived keratinocytes were the main drivers of LC renewal in intact skin 33,169. Reinforcing this observation, it has been shown that CSF-1, but not IL-34, was responsible for LC differentiation and pro-inflammatory activity in Langerhans cell histiocytosis 170. Overall, these observations suggest that both CSF-1R ligands show non-redundant roles in the lifespan of LCs 169,170.

IL-34 is highly expressed by tumor tissue in patients with melanoma, and its levels appear to depend on clinical status. An increase in IL-34 expression was observed in refractory melanomas that correlated with the expansion of CD163^+^ differentiated M2 macrophages 171. Similarly, the RUNX1/CSF-1R/IL-34 axis was responsible for the tumor rebound in BRAF inhibitor resistant melanoma. BRAF inhibitor treatment in melanoma promoted the reduction activity of the MAPK/ERK pathway, which is responsible for tumor proliferation and survival. Lower levels of the ERK pathway induced the expression of RUNX1 transcription factor, which prompted the expression and maturation of CSF-1R and IL-34. The emergence of a population resistant to the BRAF inhibitor was associated with an increase in CSF-1R and IL-34 expression, and activation of ERK1/2 and AKT signaling pathways to promote tumor survival and proliferation 172.

Overall, while IL-34 plays a positive role in LC homeostasis under healthy conditions, its overexpression is positively correlated with melanoma persistence, TAM expansion and tumor proliferation. IL-34 should be considered as an essential target for further development of therapies against melanoma.

### IL-34 in gut macrophage differentiation and intestinal disorders

Macrophages are essential both in the maintenance of healthy tissue homeostasis and in a pathogenic context. Gut macrophages originate in bone marrow. Some authors reported the existence of a heterogeneous population of macrophages in the intestine, divided into two major populations: resident macrophages and inflammatory macrophages 173,174,175. By using multi-parameter flow cytometry and lineage tracking techniques, Bain *et al.* 176 investigated the heterogeneity of macrophage populations localized in human and mouse intestines. In healthy conditions, gut macrophages underwent a continuum of differentiation from immature to fully mature subsets. Initially, macrophages were characterized by high expression of Ly6C. Then, during the maturation process, the macrophage population changed from high to low Ly6C expression, accompanied by high expression of CX3CR1, F4/80, CD64, CD11c, CD163 and CD206. In addition, mature macrophages acquired an anti-inflammatory state by presenting phagocyte activity as well as production of IL-10 176. On the other hand, in inflammatory bowel diseases (IBD), macrophage populations quickly turned to pro-inflammatory subsets characterized by high expression of Ly6C and CD14 176.

Intestine macrophage differentiation depends on activation of CSF-1R by its ligands, CSF-1 and IL-34, which are differently distributed and expressed along the intestine. CSF-1 was mainly expressed in the colon, whereas IL-34 was principally expressed in the ileum 120. Both cytokines were produced by the intestinal epithelium in response to TNF-α via the NFκB pathway. Inflammation of the gut in IBD patients was characterized by an increase in IL-34 levels produced by mononuclear cells in the lamina propria. This increase in IL-34 induced macrophage differentiation and the expression of pro-inflammatory factors TNF-α and IL-6 119. Franzè *et al.* 122 recently demonstrated that IL-34, through specific activation of the p38 MAP pathway, induced the secretion of collagen and contributed to wound healing in CD patients.

Colorectal carcinoma cells express CSF-1R and can be considered as potential targets of CSF-1R ligands 121. IL-34 induced the expression of CCL20 in a DLD1 colorectal adenocarcinoma cell line via activation of the ERK1/2 pathway and stimulation of the inflammatory response. This suggests that IL-34 participates in the crosstalk between the epithelium and immune system in inflammatory bowel diseases 121. A recent study demonstrated that IL-34 was expressed in colorectal carcinoma patient samples and induced the proliferation and invasion of colorectal cancer cells. Furthermore, DLD1 cell proliferation implied activation of the ERK1/2 signaling pathway by IL-34 through CSF-1R, and not by the CSF-1 cytokine. In fact, inhibition of IL-34 increased the sensitivity of colon tumor cells to oxaliplatin, suggesting that IL-34 may act as a pro-inflammatory and pro-tumorigenic factor in IBD disease and colorectal carcinomas 56.

### Involvement of IL-34 in the differentiation of Kupffer cells and liver diseases

Kupffer cells (KCs) are tissue-resident macrophages in the liver. KCs are responsible for the homeostasis of this organ in both healthy and pathological situations 177. Like LCs, KCs originate in the fetal liver and yolk sac. In the adult liver, the KC population is maintained by self-renewal and by bone marrow circulating monocytes 15. Depending on the microenvironment, KCs can be polarized into an M1 pro-inflammatory subtype, leading to a Th1 response, or into an M2 anti-inflammatory subtype, leading to a Th2 response. The latter is associated with inducing and maintaining immune tolerance during liver transplantation 178. Rat and human regulatory T (Treg) cells FOXP3^+^ CD4^+^ or CD8^+^ cells expressed IL-34 179. Treg FOXP3^+^ cells, the main players in immune tolerance, seemed sensitive to IL-34 178. IL-34 treatment effectively seemed to induce Treg FOXP3^+^ cell proliferation by polarizing CD14^+^ macrophages that over-expressed arginase-1 and inducible NO synthase, both implicated in the inhibition of T lymphocyte proliferation 179. These data suggest the presence of a positive feedback loop between IL-34 expression and FOXP3^+^ Tregs. Interestingly, the fact that IL-34 targeted FOXP3^+^ Tregs, which are essential for inhibiting the anti-donor immune response during allografts, suggests that IL-34 plays a potential role in transplantation therapy 179. In agreement with these data, during pregnancy, IL-34 induced macrophage polarization into an M2 state, suggesting that IL-34 may play a role in maintaining tolerance during the gestation process 64. Reinforcing the role of IL-34 in immune tolerance, Zhao *et al.* 180 showed that IL-34 treatment inhibited acute rejection in liver transplant in rats. The authors observed that IL-34 specifically induced the polarization of KCs from an M1 to an M2 status. This function was mediated by activation of the PI3K/AKT/mTOR pathway and was inhibited by rapamycin, an inhibitor of the mTOR pathways 180. Recently, Jiant *et al.* 73 reported that in zebrafish, *in vivo* ectopic expression of IL-34 in the liver or skin induced migration of macrophages to both regions. Consistent with previous studies, IL-34 may have a protective effect by mobilizing and polarizing macrophages in the liver 73.

In non-alcoholic fatty liver disease (NAFLD), a positive correlation between IL-34 levels in serum and the severity of NAFLD has been described 181. IL-34 production by liver fibroblasts was enhanced by TNF-α 181. In addition, IL-34 induced MIP3α^+^(CCL20^+^) macrophage differentiation, which promoted the production of collagen by hepatic stellate cells and subsequently the development of fibrosis 81,181. The role of IL-34 in fibrosis was recently confirmed. Soluble egg antigen (SEA) produced by schistosome egg impairs NF-κB activation in hepatic stellate cells. This inhibits TNF-α blocking the IL-34-associated liver fibrosis 182. Consequently, IL-34 may be used as an independent marker for liver fibrosis in NAFLD 181.

All these data suggest an important role for IL-34 in KC differentiation that may favor immune-tolerance during transplantation. However, as observed in other tissues, in pathogenic situations IL-34 is capable of inducing macrophage pro-inflammatory polarization with a negative impact on the severity of liver diseases.

## Macrophage as theranostic tools

**Figure 5:**
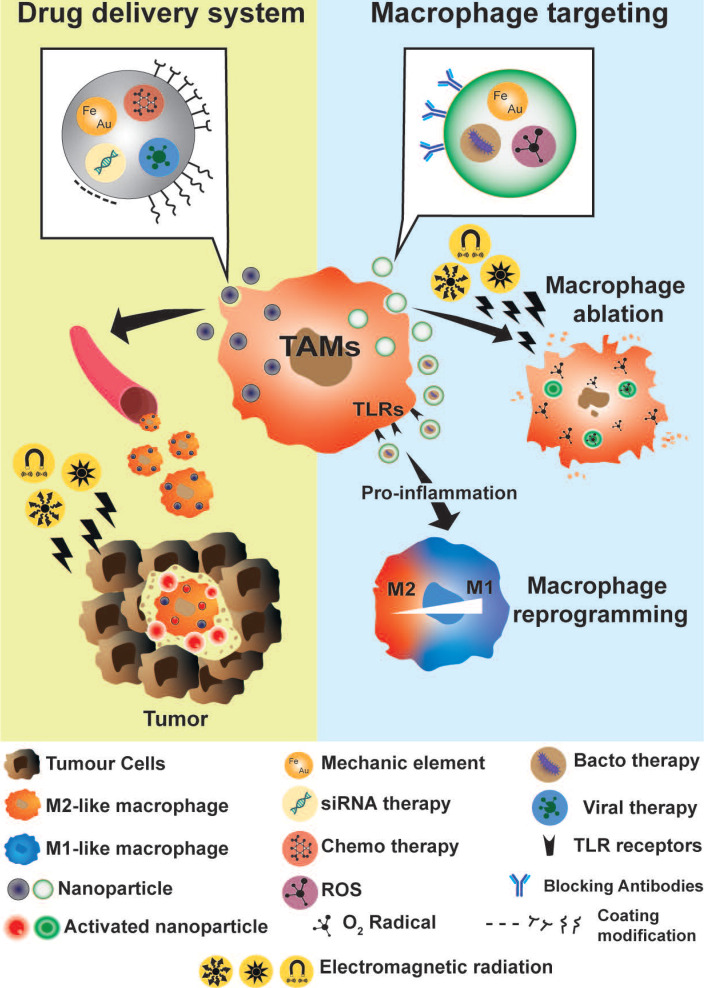
** Macrophages as a theranostic tool.** Macrophages can be used as drug delivery systems and as therapeutic targets for anti-inflammatory or anti-tumor therapy. Due to their ability to infiltrate the tumor microenvironment, macrophages can be used as drug vehicles for tumor therapy. Various types of theranostic nanoparticles were developed to simultaneously combine imaging and therapeutic approaches. Different types of electromagnetic radiations were used for functional and anatomical imaging. To favor the phagocytosis of nanoparticles, various methods of coating were proposed, including nanoparticle repolarization. Once macrophages were located in the regions of interest (inflammatory or tumor regions), these irradiation sources were also used to activate nanoparticle elements for PTT (e.g. WO, AuNRs) and delivery of chemotherapy agents (e.g. siRNA, oncolytic viruses). As macrophages markedly contribute to the development of tumors or inflammatory diseases, specific targeting of macrophages leading to their elimination or repolarization was proposed as theranostic tools. Macrophage ablation is based on PTT, ROS therapies or blocking antibodies. Macrophage reprogramming methods are based on the generation of nanoparticles with bacterial or viral elements that activate macrophage TRL signaling and induce the production of pro-inflammatory molecules which reprogram TAMs into a M1 subtype.

Due to their phagocytic and extravasation/infiltration properties, macrophages have been considered an excellent cargo system for drug delivery at specific sites of interest, as well as potential biomarkers to follow disease progression or therapeutic efficacy (**Figure 5**) 183. As previously described, macrophages also actively participate in the inflammatory process, as well as in tumor progression and development. Their biological functions identified macrophages as potential targets for anti-inflammatory and anti-tumor therapy. In recent years, many groups have focused their work on developing theranostic tools, based on macrophage bio-applications by increasing their biocompatibility, functionality, and imaging properties 6,7,183,184.

### Macrophage tracking and drug delivery systems

Characterized as non-invasive methods, electromagnetic radiation techniques are used preferentially for obtaining functional and anatomical information during macrophage associated imaging and therapy 183. They can be divided into two main groups: ionizing and non-ionizing radiations. Non-ionizing applications are based on electromagnetic radiations with low energy such as bioluminescence or fluorescence, and radio-electromagnetic waves. Optical imaging is widely used for studying sub-cellular compartments, cells, and full body. Based on light irradiation from the ultraviolet, visible, and near-infrared spectrums, optical imaging techniques have high detection sensitivity but are limited by quenching and the photo-bleaching effect. Magnetic resonant imaging (MRI) is a radiation-free and non-invasive technique based on magnetic contrast agents such as Gadolinium (III), superparamagnetic iron oxide nanoparticles (SPIO), or perfluorocarbons (PFCs), and is characterized by high spatial resolution but with very low sensitivity 185,186,187,188. Ionizing radiation such as positron emission tomography (PET) and single-photon emission computed tomography (SPECT) are based on the use of high-energy elements (radioisotopes) characterized by high-detection sensitivity (from nanomolar to picomolar) but with low spatial resolution 189,190. To obtain anatomical images, ionizing radiations are combined with another high-energy approach based on X-rays, computed tomography (CT), or non-ionizing methods such as MRI 191. To solve the different issues regarding the lack of sensitivity or spatial resolution in previous techniques, multimodal imaging was developed. These methods consist in combining ionizing and non-ionizing methods that bring high-spatial and sensitivity resolution 192,193. The accumulation of TAMs in solid tumors after chemotherapy was associated with tumor relapse and metastatic development. Early detection of TAMs can be used to predict tumor relapse and then adapt the therapeutic approach. A multimodal imaging application was developed to detect TAMs by using specific probes against the macrophage mannose receptor (MMR, or CD206), which is highly expressed by M2-like macrophages 194. Anti-CD206 antibodies were labelled with Dylight755-N-hydroxysuccinimide (NHS) ester, a near-infrared fluorescence probe (NIRF) and radiolabeled with Na^125^I, for SPECT detection. CD206 probes resulted in efficient *in vivo* tracking of M2-like macrophage infiltration in 4T1 tumor-bearing mice. Combination of NIRF and SPECT/CT CD206 imaging revealed an increase in M2-like macrophage infiltration after chemotherapy that enhanced the tumor relapse, spreading of cancer cells, and development of lung metastases. Impairment of M2-like macrophage polarization of TAMs by zoledronic acid resulted in a reduction in tumor relapse 195. These results suggested that M2-like macrophages could be used as a biomarkers to predict and adapt tumor therapy prior to tumor relapse 194.

#### Macrophages as drug delivery systems for inflamation and tumor therapies

Due to their phagocytic capacity, and their ability to both transmigrate between various tissue sites, and participate actively in inflammatory processes, macrophages were considered to be a great tool for cell-mediated drug delivery with therapeutics and imaging purposes for cancer and inflammatory diseases (**Figure 5**). Theranostics macrophage-based micro-robots were developed for tumor therapy 196. These micro-robots consisted in introducing nanoparticles of Fe_3_O_4_ (mechanic element) and docetaxel (chemotherapy element) into micro-spheres of poly-lactic-co-glycolic-acid (PLGA) that were engulfed by macrophages. Interestingly, the morphology and functionality of modified macrophages were not altered. Micro-robots were tested *in vitro* using 3D tumor spheroids, and an electro-magnetic field generator system was used to drive macrophages inside the spheroids. As expected, the viability of spheroids was compromised by the cytotoxic effect of the chemotherapy agent delivered by the micro-robots.

Poly lactic acid polymer was also used to develop biodegradable photoluminescent poly lactic acid (BPLP-PLA) nanoparticles for melanoma therapy 197. The specific anti-cancer drug for BRAF oncogene inhibition, PLX4032, was encapsulated in BPLP-PLA nanoparticles. To increase their uptake by macrophages, nanoparticles were modified by adding muramyl tripeptide (MTOP). *In vivo* and *in vitro* assays demonstrated how engineered macrophages efficiently delivered BPLP-PLA theranostic nanoparticles for targeting melanoma cells specifically, and inhibiting the associated tumor growth in a murine preclinical model 197. Nitric oxide (NO) was characterized as a potential anti-tumor therapy. However, its short lifetime *in vivo* and the negative side effects observed after systemic administration, which requires high concentrations and *in situ* distribution, has limited its therapeutic use. To counteract these limitations, a modified version of PLA nanoparticle [poly-lactic-co-glycolic acid (PLGA)] has been developed for NO delivery and tumor treatment 198. NO theranostic nanoparticles were composed of PLGA spheres packing photoNORM (photo-activated NO realising system) and Nd-UCNOPs (Nd^3+^-doped upcorventing molecules) that allowed NO delivery and photoluminescence tracking upon NIR radiation. NO-PLGA nanoparticles were loaded by phagocytosis into marrow-derived macrophages and showed no macrophage cytotoxicity. Low intensity NIR irradiation of infiltrated tumors resulted in alterations of the tumor microenvironment, and more specifically in reducing HIF-1α factor associated with tumor survival, metastasis, and angiogenesis, whereas high intensity irradiation showed high cytotoxicity in tumor cells. This macrophage-mediated NO-PLGA theranostic system paved the way for new therapeutic approaches in oncology and chronic inflammatory diseases in a controllable and localized manner 198. Based on PLGA nanoparticles, Bai *et al.* 199 developed a macrophage drug-delivery theranostic tool using tungsten oxide (WO). WO is characterized by high efficiency for photothermal therapy (PTT), low cost, and higher yields. However, WO has no fluorescence property that limits its use as a theranostic tool. WO-PLGA was implemented by introducing indocyanine green molecules (ICG), a water soluble near infrared dye. WO-ICG-PLGA nanoparticles were exposed and engulfed by macrophages that acted as vehicles to target tumor cells. This platform resulted in an excellent PTT system showing significant reduction in solid tumors *in vivo* 199.

Polyethylene glycol (PEG) polymer has been extensively exploited as another spheroid structure for producing theranostic nanoparticles for macrophage delivery 200. PEG coating increases the mass and solubility, and acts as a protective barrier to the particles of interest. On the other hand, PEG coating can be removed in the presence of albumin by a process known as de-PEGylation and can be used for nanoparticle delivery 201. Based on these properties, a hybrid lipid-PEG nanoparticle was developed for siRNA delivery and specific gene silencing in tumor tissues. Neutral lipid-PEG nanoparticles presented better gene silencing efficacy, but a lower blood circulation time and macrophage uptake than anionic formulations 202.

Small gold nanorods (AuNRs) present strong absorption in the near-infrared region (NIR) and high photothermal conversion efficiency and stability, making them suitable for photoacoustic imaging (PA) and PTT 203. In addition, they are characterized by high biocompatible and easy functionality. Surface modification of AuNRs with negative charges (Anionic-AuNRs) showed better uptake by macrophages, with less cytotoxicity than neutral or positive charged AuNRs 204. When anionic-AuNRs macrophages were inoculated into a murine tumor model, modified macrophages were localized by PA at hypoxic tumor regions. Moreover, PTT activation of anionic-AuNRs macrophages resulted in tumor ablation and improvement of mouse survival 204. To increase the potential of AuNRs as anti-tumor therapy, macrophage drug delivery systems were implemented by introducing chemotherapy agents into nanoparticles. A synergistic platform that combined PTT (AuNRs) and chemotherapy was recently developed 205. Doxorubicin (Dox) anti-tumor drugs were introduced into liposomes (LP) using the thin-film hydration method 206. AuNRs plus Dox-LP- were introduced into macrophages using the exposure and innate phagocytosis process. The small size of AuNRs (7nm) and LP (145nm) favored the uptake of nanoparticles by macrophages. Irradiation of nanoparticles by NIR induced photothermal conversion by AuNRs that generated thermal degradation of LP and delivery of Dox. *In vitro* and *in vivo* experiments using 4T1 breast tumor cells showed reduced tumor volume after NIR irradiation of Dox-LP-AuNRs 205.

Another approach for macrophage drug delivery bio-applications implied using macrophage surface modification to anchor specific anti-tumor drugs. CD14 and Toll-like receptor 4 (TLR4) are common receptors expressed at the surface of macrophages. These receptors have a high affinity for LPS expressed in gram-negative bacteria. C-C chemokine receptor type 2 (CCR2) is also expressed by macrophages in response to the production of chemoattractant protein-1 (CCL2) expressed by tumor cells. Taking advance of these receptors, engineered macrophages were developed to deliver Dox (LPS-membrane anchored macrophages encapsulating Dox, LMs-Dox) 207. This delivery platform implied the construction of LPS-anchored macrophages (LM) by co-incubation of LPS and M1 subtype macrophages. The LMs-Dox system inhibited primary lung cancer cell growth and proliferation in both *in vitro* and *in vivo* assays. Moreover, the LMs-Dox system produced a synergistic therapeutic effect by inducing TNF-α expression in TAMs. High levels of TNF-α generated a cytotoxic effect in tumor cells and inhibited tumor growth 207. To prevent nanoparticles being recognized by the immune system, iron oxide nanoparticles encapsulated in macrophage membrane has been proposed for photothermal tumor therapy 208. A variety of cell membrane-coated particles has been developed thanks to their ability to keep desirable features from the source cells, such as cell-cell adhesion 209. Macrophage membranes are able to interact with tumor cells. Biomimetic iron oxide nanoparticles have presented high biocompatibility, immune evasion, and tumor attraction in both *in vitro* and *in vivo* assays. Due to their magnetic and photothermal properties, biomimetic iron oxide nanoparticles resulted in a significant slowdown in tumor progression in a murine immunodeficient model of breast cancer treated with photothermal therapy 208. Overall, theranostic tools based on cell membrane-coating have shown promising biomedical applications. Using similar approaches, a PTT-chemotherapy theranostic platform of Au-nanocages encapsulating Dox that can coat cancer cell membranes has been described 210. These nanoparticles displayed high load anticancer drugs, high biocompatibility, immune escape, prolonged blood circulation, absence of an *in vivo* inflammatory reaction and remarkable tumor targeting properties. Once localized in regions of interest, they exhibited strong photothermal conversion capacity upon NIR irradiation and on-demand Dox release, revealing highly efficient tumor ablation with no side effects 210.

The property of TAMs to be attracted to the hypoxic tumor microenvironment was exploited to deliver therapeutic adenovirus in prostate tumor regions 211. Macrophages were genetically modified by co-transduction of a hypoxia-regulated E1A/B element and an E1A/B-dependent adenovirus. Ad[I/PPT-E1A] specifically targeted prostate tumor cells and showed highly oncolytic activity 212. Infiltration of modified macrophages in hypoxic regions of prostate tumors induced the expression of E1A/B proteins, which triggered transduction of viral particles and adenovirus delivery. The specificity of Ad[I/PPT-E1A] for prostate-specific promotor elements in prostate tumor cells favors the infection of tumor cells, replication of the virus, and destruction of tumor cells, which resulted in a lasting anti-tumor effect 211. Treatment with Ad[I/PPT-E1A] macrophage delivery therapy after chemotherapy (docetaxel) or tumor irradiation resulted in reduced primary prostate tumor regrowth and metastasis in mouse models. Moreover, combined treatment showed a significant increment of lifespan in tumor-bearing mice 213. Recently, mathematical modeling demonstrated that macrophage viral therapy resulted in maximum therapeutic effect when it was introduced right after irradiation treatment 214. Authors have indicated that radiotherapy may increase the secretion of chemoattractants by tumor and dead cells, which may stimulate the migration of Ad[I/PPT-E1A] macrophages to specific tumor regions. Once localized in hypoxic areas, engineered macrophages release the virus, which could infect tumor cells and exert oncolytic activity.

Overall, the ability of engineered macrophages to access particular pathogenic niches to exert control over the immune system has important advantages for the development of new therapeutic tools for treating inflammatory and oncologic diseases. While some of these techniques are in the proof-of-concept phase in murine models, preliminary results have demonstrated the potential of these theranostic tools for reducing tumor progression and metastasis, together with other biomedical applications.

### Targeting macrophages using theranostic applications

As precursors and enhancers of the inflammatory process and tumor development, macrophages were considered to be ideal target for theranostic therapies. Macrophage depletion and macrophage polarization were the two main approaches used. As with macrophage-drug delivery theranostic applications, the innate phagocytic properties of macrophages was highly deployed to develop macrophage depletion theranostics (**Figure 5**).

#### Macrophage ablation

Due to their non-invasive and powerful imaging properties, magnetic resonant probes were used for macrophage depletion. Manganese ferrite nanoparticles (MFNPs) are characterized by high magnetic susceptibility 215. To favor their blood circulation, low toxicity and macrophage uptake, the surface of MFNP nanoparticles was coated with the surfactant polysorbate 80. Three different types of polysorbate 80 coatings were generated with different charges: anionic, cationic, or neutral. Although the three versions showed similar physical properties, cationic MFNPs resulted in the highest uptake, and cytotoxicity was observed when murine macrophages (RAW264.7 cells) were exposed to cationic MFNPs. These results were explained by the negative charges of the macrophage membranes, which favored their interaction with cationic MFNPs. This suggested that the polarity of the nanoparticles should be considered as a key element for the development of macrophage-based theranostic applications 216.

During tumor development, cancer cells can initiate the metastatic process by inducing macrophage recruitment and proliferation, which leads to the formation favorable local niches for cancer cell proliferation. The CD137 signaling pathway plays an essential role in promoting macrophage migration. CD137 is a member of the TNF receptor superfamily that is mainly expressed by immune cells. The CD137 ligand (CD137L) is expressed by cancer cells and interacts with CD137 at the cell surface of macrophages, and triggers the activation of signaling pathways that control cell migration. Jiang *et al.* 217 recently developed a liposome nanoparticle that carries a specific anti-CD137 blocking antibody. Transwell assay showed the ability of the anti-CD137 to block the migration properties of RAW264.7 mouse macrophages induced by mouse breast cancer cells. Anti-CD137 antibodies were encapsulated in liposome nanoparticles, coated with an anti-F4/80 antibody that targets the F4/80 marker expressed by M1 and M2 mouse macrophages. Treatment of mice bearing 4T1 breast cancer cells with NP-αCD137 Ab-F4/80 nanoparticles resulted in *in vivo* inhibition of bone and lung metastases, suggesting that NP-αCD137 Ab-F4/80 nanoparticles blocked the CD137 signaling and compromised the migration property of macrophages to tumor areas 217.

Photodynamic therapy (PDT) is another technique widely used for cell depletion. In the presence of oxygen, light irradiation of a photosensitive molecule induces the generation of reactive oxygen species (ROS) and subsequently cell lethality 218. Ben-Nun *et al.* 219 developed a PDT theranostic application for macrophage ablation. Cathepsins are highly expressed by TAMs and are associated with tumor progression 220. Small molecules quenched activity-based probes (qABPs) with specific sequences against cathepsins, which were coupled to photosensitizers and used to target TAMs in a murine breast cancer model. Activation of photosensitizers by light allowed image detection of TAMs in tumor regions and specific macrophage ablation by ROS cytotoxic activity 219. PDT was also used to develop another photosensitizer probe (TPE-Man) by targeting a mannose receptor (CD206) that is overexpressed by TAMs. This theranostic probe consisted in two α-mannose moieties linked to a red-emissive and aggregation-induced emission-active (AIE) photosensitizer core. The interaction between the α-mannose moieties and the mannose receptor CD206 induced the internalization of the probe into the macrophage cytoplasm. *In vitro* studies of TAMs containing the TPE-Man probe showed that, while incubation in dark conditions did not compromise TAMs viability, the exposure of TAMs to white light irradiation induced photodynamic therapy with the production of ROS species and subsequently efficient TAM ablation 221.

As with TAM ablation, theranostic applications targeting macrophages in chronic inflammatory diseases have been developed. Isoliquiritigenin (ISL) is a flavonoid obtained from licorice that has demonstrated that it potentially attenuates osteoclastogenesis 222. However, ISL is characterized by poor solubility, and short half-life and bioavailability. To circumvent these problematic properties, mesoporous silica nanoparticles (MSNs) were engineered to deliver ISL 223. MSN nanoparticles proved to be excellent biocompatible vehicles for flavonoid delivery. MSNs-ISL induced *in vitro* inhibition of the osteoclastogenesis process by inhibiting the RANKL-signaling pathway. Moreover, treatment of mouse calvaria with MSNs-ISL nanoparticles induced *in vivo* reduction in osteoclast activity and relieved inflammation-associated calvarial bone destruction 223.

Macrophage ablation techniques result in atractive tools for reducing the negative effect of TAMs and pro-inflammatory macrophages. However, improvements in these techniques, allowing them to target specific macrophage subtypes are required.

#### Macrophage reprogramming

Generally, TAMs are characterized by the acquisition of M2-like macrophage polarization, which facilitates tumor progression, angiogenesis, and metastasis. However, depending on the microenvironment, TAMs can also polarize to the M1 subtype, which induces local inflammation leading to the anti-tumor response 90. The ability of macrophages to switch from one subtype to another one has been used to develop theranostic anti-cancer therapies.

Solid tumors are characterized by the generation of a hypoxic and anoxic microenvironment that can result in a perfect niche for facultative or obligatory bacteria such as *Clostridium*, *Escherichia*, *Listeria* and *Salmonella*. This opened the door to the development of bacterial cancer therapies. The attenuated *Salmonella typhimurium* strain was used to develop an anti-cancer therapy 224. *S. typhimurium* was genetically modified to overexpress and secrete a heterologous bacterial flagellin (FlaB) which triggers the inflammatory response via the Toll-like receptor 5 (TLR5) signaling pathway. Bacterial infection of a murine adenocarcinoma model induced a dual inflammatory response by activating the TLR4 signaling pathway (due to bacterial colonization of tumor regions) and the TLR5 signaling pathway (after local secretion of FlaB). Analysis of the macrophage population showed an M2-to-M1 switch, coupled with a high secretion of pro-inflammatory factors and cytotoxic agents. These changes in tumor microenvironment induced a reduction of tumor growth 224. TLR3 is another member of the TLR family expressed by macrophages that mediates the antiviral response by recognizing viral dsRNA. Polyinosinic-polycytidylic acid (PIC), an agonist of TLR3, was used to induce TAM polarization into an M1 anti-tumor subset 225. Iron oxide nano-carrier particles (ferumoxytol®, FMT) are biocompatible, as well as being low cytotoxicity nanoparticles that can be imaged by MRI and used for hyperthermia therapy, integrating diagnostic and therapeutic functions. PIC was complexed to an amino-modified FMT to obtain the FMT-NH_2_-PIC nanocomposite (FP-NPs). *In vitro* and *in vivo* studies using murine melanoma models showed that FP-NP treatment induced M1 polarization of TAMs with anti-tumor properties, as well as metastatic melanoma regression 225. Resiquimod (R848), a potent TLR7/8 agonist, was used to develop β-cyclodextrin nanoparticles (CDNPs-R848) that induced TAM reprogramming with reduced tumor growth and protection against tumor relapse 226. However, CDNPs-R848 produced systemic side effects in mouse models due to fast release of R848. Chemical modification of R848 with adamantane (R848-Ad@CDNP) resulted in reduced systemic toxicity without altering the capacity for TAM reprogramming and tumor regression 227. Low-molecular-weight hyaluronic acid (LMWHA) also promotes M1 macrophage polarization 228. Based on LMWHA, multifunctional nanoparticles were developed to combine macrophage reprogramming and modulation of the microenvironment for cancer therapy 229. Mesoporous Prussian blue nanoparticles (MPB) were coated with LMWHA and used as a cargo for sonosensitizer hematoporphyrin monomethyl ether (HMME). *In vitro* assays demonstrated the ability of LMWHA-MPB/HMME to induce TAM polarization into an M1 anti-tumor subtype. Apart from the cargo role, MPB was used as an NIR image probe to demonstrate *in vivo* accumulation of LMWHA-MPB/HMME in tumor sites. Ultrasound irradiation induces the production of H_2_O_2_ from HMME and MPB acts as a catalyst of hydrogen peroxide (H_2_O_2_) to produce O_2_. *In vivo* experiments using 4T1 tumor-bearing mice showed that ultrasound irradiation of LMWHA-MPB/HMME induced both the production of O_2_ and the transformation of the tumor microenvironment 229.

Overall, these results demonstrated the potential of multifunctional theranostic applications to control macrophage polarization and the tumor microenvironment. However, such applications still need to be improved for controlling the switch of one macrophage population to another subtype in a reproducible manner and without generating limited side effects.

## Conclusion

Final macrophage differentiation relies on the action of specific growth factors and their specific receptors. The “twin” cytokines CSF-1 and IL-34 play an essential role in the differentiation and proliferation of non-resident and tissue-resident macrophages 24,25. Both cytokines share a common receptor, CSF-1R, and their binding to the receptor activates the same signaling pathways. However, the similarities between CSF-1 and IL-34 ended there. Each cytokine presented specific domains of interaction with CSF-1R and the nature of these interactions generated differential activation of the receptor 36. IL-34 induced a strong but transitory phosphorylation of CSF-1R tyrosine residues, followed by rapid downregulation of the receptor 38. These differences in CSF-1R activation generated a variety of downstream signaling pathway activations and, as a consequence, a wide range of biological functions in macrophages and other cell types. Adding another layer of complexity, CSF-1 and IL-34 were able to generate a functional heterodimer 48, and both cytokines could be present in several isoforms 57,58. Moreover, IL-34 bound to RPTP-ζ and syndecan-1 receptors with particular bioactivities 52,53. Overall, these studies suggest that activation of CSF-1R by its ligands involves a complex network of specific interactions leading to specific biological activities. New studies and approaches are now mandatory for deciphering how each particular cytokine regulates the different signaling pathways and their roles in macrophage differentation. The existence of new heterodimers, isoforms or receptors cannot be excluded.

Under normal physiology conditions, and depending on their microenviroment, circulating monocytes could be differentiated by CSF-1 and IL-34 into specific non-resident macrophages with a “pro-inflammatory” M1 phenotype or an “anti-inflammatory” M2 phenotype. Treating monocytes with IL-34 primarily induced macrophage differentiation into an M2 phenotype 63. The ability of each cytokine to induce different macrophage diferentation was also oberved in otherr species, such as birds 47, fish 67,68,69,73 and frogs 66, contributing to and offering new potential animal models for advancement in knowledge of both cytokines and macrophage differentiation.

CSF-1 and IL-34 are also important for the development, differentiation and proliferation of tissue-resident macrophages. In bone, IL-34 is able to regulate the adhesion, differentiation and proliferation of osteoclast precursors, and may replace CSF-1 during osteoclastogenesis 43. In the CNS, IL-34 is necessary for maintaining microglia during adulthood, as well as for the proper development of brain areas associated with high IL-34 expression (e.g cortex) 155 and the retinal microglia population 156. In the skin, IL-34 has shown that it is essential for the development of LCs in the skin rudiment, the differentiation/proliferation of LCs in the dermis, while its absence compromised the presence of LCs in the skin. Moreover, IL-34 is essential for the renewal of LCs in intact skin 20,33,30,169. IL-34 was also important for macrophage differentiation in other organs, such as the gut, kidneys, spleen 34,68 and liver 178. The specific functions of IL-34 in most of these tissues remains unclear and the use of conditional knockout mice can help to clarify those funtions. Recent progress in single cell omics and organoid techniques may be helpful for dissecting the function of human IL-34 and the molecular cytokine-specificity of each subset of IL-34-derived macrophages, from non-residient to tissue-resident populations.

Depending on the tissue and microenviroment, the cytokine IL-34 has a positive or negative role and can be considered the Dr Jekyll and Mr Hyde cytokine. On the one hand, IL-34 acts as a beneficial factor with high potential as a therapeutic molecule. In the CNS, IL-34-derived microglia have a neuroprotective function by favoring neural myelination and survival in AD 154,162. In retinal disease, IL-34 retinal microglia protect the retinal pigment epithelium from damage 156. Secretion of IL-34 by neurons in prion infections helps microglia proliferation and neuroprotection 20. Moreover, IL-34 induces microglia resistance to West Nile virus infection and the same effects have been observed in other viral infections, such as HIV-1 76 or FV3 87. In addition, IL-34 is associated with inducing and maintaining immune tolerance in liver transplantations, as well as during pregnancy 64. These data suggest that IL-34 can be used as a powerful tool for improving graft generation and allograft tolerance.

On the other hand, as a Mr. Hyde cytokine, IL-34 can be considered a good theraupeutic target against a wide range of diseases, from virus infections such as HCV or HBV, to autoimmune diseases such as RA, systemic sclerosis, systemic lupus erythematous, Sjogren's syndrome 139 and inflammatory bowel diseases, to cancer-like osteosarcoma, lung cancers, melanoma, colorectal cancer, ovarian tumors, prostate cancer 97 and hepatocellular carcinoma 100,101. In the majority of these conditions, authors have reported that high levels of IL-34 in serum or tissue fluids correlated with poor prognosis and survival, and suggested using IL-34 as an indicator of disease grade 230,231. Furthermore, in cancer, IL-34 has been described as a pro-tumorigenic factor in a variety of tumors. IL-34 favored tumor cell proliferation and metastasis by promoting IL-34-derived macrophage extravasation and polarization into a TAM M2 phenotype. In most of the tumors, high levels of IL-34 and TAMs correlated with poor prognosis and survival. However, there were some exceptions, such as in the breast luminal B and HER2 tumor subtypes, where high levels of IL-34 were associated with better survival and good prognosis. Therapies against TAMs have been developed in recent years. Reducing and repolarising TAM macrophages has been shown to be a promising therapy 97,232,233.

In the past few years, most strategies against the pathogenic effects of CSF-1/CSF-1R and IL-34/CSF-1R have focused on CSF-1R. However, due to CSF-1R being involved in multiple biological processes, specific tumor therapies against CSF-1R have generated undesirable side effects 234. This has brought other actors in the complex to the attention of the scienctific community, notably its ligands CSF-1 and IL-34 235,236,237. Macrophage-based tools are powerful theranostic approaches in oncology, inflammatory and infectious diseases 5,7,184,238. In the present review we have discussed the prominent role of IL-34 in macrophage differentation and its positive or negative effects in healthy and pathogenic situations. Targeting IL-34 and not the CSF-1R could bypass the undesirable effects related to the other ligand, CSF-1. Theranostic applications include specific delivery of recombinant IL-34 in patients affected by diseases where IL-34 has been described as a protective or beneneficial molecule, e.g. CNS diseases, viral infections or liver transplantations. IL-34 may be targeted to induce macrophage reprograming in diseases where pro-inflammatory CSF-1 macrophages display a pathogenic role. Moreover, generating specific blocking molecules or antibodies against IL-34 could be loaded and local or systemic delivery could be obtained by macrophages engineered for specific zones where IL-34 expression has a negative role, e.g. autoimune diseases or tumors, alone or in combination with other molecules, such as chemotherapy or PTT agents, for more efficient treatment. Further studies are needed to evaluate the effects and implications of these new IL-34 therapies. IL-34 must be considered as a potential theraupetic target as well as an interesting theraupeutic tool in health issues.

## Figures and Tables

**Table 1:** Kinetic properties of CSF-1 and IL-34 binding to CSF-1R

Proteins	Binding characteristic	KD	K_on_	K_off_
CSF-1/CSF-1R	hydrophilic	1 pM	6.29 × 10^7^ s-^1^ M^-1^	6.55 × 10^-5^ s^-1^
IL-34/CSF-1R	hydrophobic	34 pM	1.7 × 10^7^ s-1 M^-1^	6.03 × 10^-4^ s^-1^

K_D_: equilibrium dissociation constant; K_on_: association rate constant; K_off_: dissociation rate constant. Data from 25.

**Table 2:** IL-34 and autoimmune diseases

Diseases	Major finding related to IL-34	References
Bowel Autoimmune Diseases	High IL-34 expression levels in lamina propria compartments in Crohn's disease (CD) and Ulcerative colitis (UC); IL-34 enhances inflammatory response by up-regulation of TNF-α and IL-6 factors	119
	High expression levels of IL-34 in CD than in UC, with IL-34 mainly expressed in the Ileum; TNF-α induces IL-34 production by epithelial cells.	120
	IL-34 participates in the cross talk between epithelial and immune cells by induction of CCL20 chemokine expression	121
	IL-34 is highly produced in the fibrotic gut of CD patients and contributes to collagen production by enhancing COL1A1 and COL3A1 expression in a p38MAP kinase-depending mechanism	122
Multiple Sclerosis (MS)	IL-34 acts as a neuroprotective factor by inducing microglia differentiation with anti-inflammatory properties	123
	Il-34 expression by neurons contributes to the re-establishment of tight junctions and blood brain barrier by epithelial cells; IL-34 induces microglia differentiation with anti-inflammatory properties	124
	IL-34 levels do not change in relapsing-remitting MS	125
	IL-34 induces neuroprotection by expansion of CD11c^+^ microglia population via CD115	126
	IL-34 expression is down regulated in cerebrospinal fluids in MS affected patients	127
	In children, Vitamin D partially induces IL-34 expression by neurons conferring neuroprotection against MS	128
Psoriasis	High levels of IL-34 in serum of patients affected by psoriatic Arthritis correlate with high levels of osteoclast precursors and poor prognosis	129
Rheumatoid Arthritis (RA)	IL-34 is highly expressed by synovial fibroblast of RA affected patients; IL-34 expression is induced by pro-inflammatory factors IL-1β and TNF-α; High levels of IL-34 correlate with severity and poor prognosis of RA	111
	High levels of IL-34 in synovial fluids of RA patients; IL-34 induces osteoclast differentiation in RA; TNF-α induces IL-34 expression by synovial fibroblast via NFκB and JNK pathways	130
	IL-34 high levels in serum and synovial fluid of RA patients; IL-34 induces the expression of pro-inflammatory factor IL-17 by circulating mononuclear cells	131
	High levels of IL-34 in serum of RA patients positively correlate with IL-6, RANKL and anti-cyclic citrullinated peptide (CCP) antibody levels	132
	High levels of IL-34 in serum and synovial fluids positively correlate with rheumatoid factors (RF), current smoking, erythrocyte sedimentation rate (ESR) and C-reactive protein levels; IL-34 as an independent risk factor for radiographic progression of RA	133
	High levels of IL-34 in serum of RA patients in stage III of hand R-ray score; IL-34 levels positively correlate with increase of pro-inflammatory factors IL-6, IL-8, MMP-3 and C-reactive protein	134
	Treatment with TNF-α antagonist reduces levels of IL-34 after 3 months of treatment and correlates with good prognosis of RA	135
	Simultaneous inhibition of M-CSF and IL-34 cytokines decrease pathology symptoms in RA mouse models and humans	136
	High levels of IL-34 in serum and synovial fluids of RA patients; IL-34 enhances synovial fibroblast apoptosis resistance by production of miR-21 via STAT3 signaling pathway activation	137
	BMP2 and TGF-β acts as controllers of inflammatory process in RA by inhibition of IL-34 expression in synovial fibroblast	115
	High levels of IL34 in serum of RA patients positively correlate with C-reactive protein, ESR, RF and anti-CCP antibody; IL-34 induces the expression of IL-6 cytokine and subsequently promotes Th17 production	113
	IL-34 plays an essential role in the immune cell cross talk during RA; IL-34/CD115 complex stimulates the expression of ROS in THP-1 cells, inducing IL-6 secretion and Th17 production	116
	IL-34 participates in the establishment of RA in mice by induction of proliferation, migration and transformation of circulating fibrocytes in fibroblast-like synovial cells in affected joints	112
	High levels of IL-34 in serum of RA patients positively correlate with RANKL, DAS28-ERS, C-reactive protein, RF and bone erosion score; IL-34 levels can be used as a predictor of bone erosion	118
	IL-34 participates in local joint destruction and osteoporosis during RA by induction of RANKL expression and inhibition of OPG, partially mediate by IL-17, in sinoviocytes fibroblast and circulating monocytes	117
	IL-34 may participate indirectly in angiogenesis process in RA by induction of VEGF and HIF-1α factors secretion in RA circulating monocytes	138
	IL-34 modulates the proliferation and migration of synoviocytes fibroblast in RA	114
Sjogren Syndrome (pSS)	IL-34 expression correlates with expansion of pro-inflammatory CD14^bright^ CD16^+^ monocytes in salivary glands; IL-34 acts as a pathogenic factor in pSS	139
	High levels of IL-34 in serum of pSS patients are positively associated with levels of RF, IgG and γ-globulin; IL-34 induces hyper-activation of B cells and antibodies production	140
Systemic Lupus Erythematosus (SLE)	High levels of IL-34 in serum of children with SLE correlate with high SLE Disease Activity Index (SLEDAI), anti-double-stranded DNA antibody (anti-sdDNA) and C-reactive protein	141
	IL-34 levels are detectable in serum of SLE affected patients and correlate with SLEDAI and high IgG; IL-34 as a potential disease activity marker for SLE	142
	High levels of IL-34 in serum and urine correlate with poor prognosis of SLE patients; IL-34 expression is associated with high expression of CD115 and PTP-ζ and induces differentiation and accumulation of intrarenal macrophages that favors tubular epithelial cell apoptosis	143
	High levels of IL-34 in serum of children with SLE correlate with high SLEDAI, anti-sdDNA and C-reactive protein, with a more aggressive effect that adult SLE	144
	High levels of IL-34 in serum of patients affected by lupus nephritis and correlate with SLEDAI, anti-sdDNA and C-reactive protein; IL-34 can be used as surrogate marker for early detection of lupus nephritis diseases	145
Systemic Sclerosis (SS)	High levels of IL-34 in serum of SS patients correlate with expansion of M2 and Th17 macrophages and severity of interstitial lung disease	146

## Abbreviations

AD: Alzheimer disease
AuNRs: Gold nanorods
BPLP-PLA: Biodegradable photoluminescent poly lactic acid
CCR: C-C chemokine receptor
CNS: Central nervous system
CRC: Colorectal cancer
CSF-1/-2: Colony Stimulating Factor-1/-2
CT: Computed tomography
Dox: Doxorubicin
ERK1/2: Extracellular signal Regulated protein Kinases 1 and 2
FAK: Focal Adhesion Kinase
FDC: Follicular dendritic cells
FMT: Ferumoxytol®
FV3: Frog virus 3
G-CSF: Granulocyte Colony Stimulating Factor
GRP: 78-KDa glucose-regulated protein
HBV: Hepatitis B virus
HCC: Hepatocellular carcinoma
HCV: Hepatitis C virus
HFRS: Hemorrhagic Fever Renal Syndrome
HIV-1: Human immunodeficiency virus-1
HMME: Hematoporphyrin monomethyl ether
IAV: Influenza A virus
IBD: Inflammatory bowel diseases
ICG: Indocyanine green
IDE: Insulin degrading enzyme
IFN-γ: Interferon gamma
IL: Interleukin
ILD: Interstitial lung disease
ISL: Isoliquiritigenin
JAK: Janus Kinase
JNK: c-JUN N-terminal Kinase
KCs: Kupffer cells
LCs: Langerhans cells
LMWHA: Low-molecular-weight hyaluronic acid
LP: Liposomes
LPS: Lipopolysaccharides
MFNPs: Manganese ferrite nanoparticles
MM: Multiple myeloma
MMR: Macrophage mannose receptor
MPB: Mesoporous Prussian blue
MRI: Magnetic resonant imaging
MS: Multiple sclerosis
MSNs: Mesoporous silica nanoparticles
MTOP: Muramyl tripeptide
NAFLD: Non-alcoholic fatty liver disease
NFκB: Nuclear Factor kappa Beta
NIRF: Near-infrared fluorescence
NO: Nitric oxide
PA: Photoacoustic
PDT: Photodynamic therapy
PEG: Polyethylene glycol
PET: Positron emission tomography
PFCs: Perfluorocarbons
PIC: Polyinosinic-polycytidylic acid
PI3K: PhosphoInositide 3-Kinase
PLGA: Poly-lactic-co-glycolic-acid
PNS: Peripheral nevous system
PRRs: Pattern recognition receptors
PTP-ζ: Protein-Tyrosine Phosphatase-zeta
PTT: Photothermal therapy
RA: Rheumatoid arthritis
RANKL: Receptor Activator of Nuclear Factor kappa-B ligand
ROS: Reactive oxygen species
SEA: Soluble egg antigen
SLE: Systemic lupus erythematosus
SPECT: Single-photon emission computed tomography
SPIO: Superparamagnetic iron oxide nanoparticles
SSc: Systemic sclerosis
STAT3: Signal Transducer and Activator of Transcription 3
TAMs: Tumor-associated macrophages
TME: Tumor microenvironment
TGF-β1: Transforming growth factor β1
TLR: Toll-like receptor
TNF-α: Tumor necrosis factor alpha
WNV: West Nile virus
WO: Tungsten oxide

## References

[1] Jaumouillé V, Grinstein S (2016). Molecular Mechanisms of Phagosome Formation. Microbiol Spectr.

[2] Biron CA (2016). Chapter 4 - Innate Immunity: Recognizing and Responding to Foreign Invaders-No Training Needed. Third Edition ed. Elsevier Inc.

[3] Vannella KM, Wynn TA (2017). Mechanisms of Organ Injury and Repair by Macrophages. Annu Rev Physiol.

[4] Wynn TA, Chawla A, Pollard JW (2013). Macrophage biology in development, homeostasis and disease. Nature.

[5] Patel SK, Janjic JM (2015). Macrophage Targeted Theranostics as Personalized Nanomedicine Strategies for Inflammatory Diseases. Theranostics.

[6] He H, Ghosh S, Yang H (2017). Nanomedicines for Dysfunctional Macrophage-Associated Diseases. J Control Release.

[7] Zanganeh S, Spitler R, Hutter G, Ho JQ, Pauliah M, Mahmoudi M (2017). Tumor-associated Macrophages, Nanomedicine and Imaging: The Axis of Success in the Future of Cancer Immunotherapy. Immunotherapy.

[8] van Furth R, Cohn ZA (1968). The Origin and Kinetics of Mononuclear Phagocytes. J Exp Med.

[9] Davies LC, Taylor PR (2015). Tissue-resident macrophages: Then and now. Immunology.

[10] Perdiguero EG, Geissmann F (2016). The development and maintenance of resident macrophages. Nat Immunol.

[11] Franken L, Schiwon M, Kurts C (2016). Macrophages: Sentinels and regulators of the immune system. Cell Microbiol.

[12] Hoeffel G, Ginhoux F (2018). Fetal monocytes and the origins of tissue-resident macrophages. Cell Immunol.

[13] Stremmel C, Schuchert R, Wagner F, Thaler R, Weinberger T, Pick R (2018). Yolk sac macrophage progenitors traffic to the embryo during defined stages of development. Nat Commun.

[14] Okabe Y (2018). Molecular control of the identity of tissue-resident macrophages. Int Immunol.

[15] Hashimoto D, Chow A, Noizat C, Teo P, Beasley MB, Leboeuf M (2013). Tissue-resident macrophages self-maintain locally throughout adult life with minimal contribution from circulating monocytes. Immunity.

[16] Shapouri-Moghaddam A, Mohammadian S, Vazini H, Taghadosi M, Esmaeili SA, Mardani F (2018). Macrophage plasticity, polarization, and function in health and disease. J Cell Physiol.

[17] Hume DA, Irvine KM, Pridans C (2019). The Mononuclear Phagocyte System: The Relationship between Monocytes and Macrophages. Trends Immunol.

[18] Dai XM, Ryan GR, Hapel AJ, Dominguez MG, Russell RG, Kapp S (2002). Targeted disruption of the mouse colony-stimulating factor 1 receptor gene results in osteopetrosis, mononuclear phagocyte deficiency, increased primitive progenitor cell frequencies, and reproductive defects. Blood.

[19] Sakagami T, Uchida K, Suzuki T, Carey BC, Wood RE, Wert SE (2009). Human GM-CSF Autoantibodies and Reproduction of Pulmonary Alveolar Proteinosis. N Engl J Med.

[20] Wang Y, Szretter KJ, Vermi W, Gilfillan S, Rossini C, Cella M (2012). IL-34 is a tissue-restricted ligand of CSF1R required for the development of Langerhans cells and microglia. Nat Immunol.

[21] Meshkibaf S, William Gower M, Dekaban GA, Ouk Kim S (2014). G-CSF preferentially supports the generation of gut-homing Gr-1 high macrophages in M-CSF-treated bone marrow cells. J Leukoc Biol.

[22] Duplomb L, Baud'huin M, Charrier C, Berreur M, Trichet V, Blanchard F, Heymann D (2008). Interleukin-6 Inhibits Receptor Activator of Nuclear Factor κB Ligand-Induced Osteoclastogenesis by Diverting Cells into the Macrophage Lineage: Key Role of Serine727 Phosphorylation of Signal Transducer and Activator of Transcription 3. Endocrinology.

[23] Yin Z, Ma T, Lin Y, Lu X, Zhang C, Chen S, Jian Z (2018). IL-6/STAT3 pathway intermediates M1/M2 macrophage polarization during the development of hepatocellular carcinoma. J Cell Biochem.

[24] Stanley ER, Chen DM, Lin HS (1978). Induction of macrophage production and proliferation by a purified colony stimulating factor. Nature.

[25] Lin H, Lee E, Hestir K, Leo C, Huang M, Bosch E (2008). Discovery of a cytokine and its receptor by functional screening of the extracellular proteome. Science.

[26] Alothaimeen T, Seaver K, Mulder R, Gee K, Basta S (2020). Granulocyte/Macrophage Colony-Stimulating Factor-Derived Macrophages Exhibit Distinctive Early Immune Response to Lymphocytic Choriomeningitis Virus Infection. Viral Immunol.

[27] Guilbert LJ, Stanley ER (1980). Specific Interaction of Murine Colony-Stimulating Factor With Mononuclear Phagocytic Cells. J Cell Biol.

[28] Yeung YG, Jubinsky PT, Sengupta A, Yeung DCY, Stanley ER (1987). Purification of the colony-stimulating factor 1 receptor and demonstration of its tyrosine kinase activity. Proc Natl Acad Sci U S A.

[29] Stanley ER, Chitu V (2014). CSF-1 receptor signaling in myeloid cells. Cold Spring Harb Perspect Biol.

[30] Ginhoux F, Tacke F, Angeli V, Bogunovic M, Loubeau M, Dai XM (2006). Langerhans cells arise from monocytes *in vivo*. Nat Immunol.

[31] Kondo Y, Duncan ID (2009). Selective reduction in microglia density and function in the white matter of colony-stimulating factor-1-deficient mice. J Neurosci Res.

[32] Ginhoux F, Greter M, Leboeuf M, Nandi S, See P, Gokhan S (2010). Fate mapping analysis reveals that adult microglia derive from primitive macrophages. Science.

[33] Greter M, Lelios I, Pelczar P, Hoeffel G, Price J, Leboeuf M (2012). Stroma-Derived Interleukin-34 Controls the Development and Maintenance of Langerhans Cells and the Maintenance of Microglia. Immunity.

[34] Nakamichi Y, Mizoguchi T, Arai A, Kobayashi Y, Sato M, Penninger JM (2012). Spleen serves as a reservoir of osteoclast precursors through vitamin D-induced IL-34 expression in osteopetrotic op/op mice. Proc Natl Acad Sci U S A.

[35] Gow DJ, Garceaua V, Kapetanovica R, Sestera DP, Ficib GJ, Shelly JA (2012). Cloning and expression of porcine Colony Stimulating Factor-1 (CSF-1) and Colony Stimulating Factor-1 Receptor (CSF-1R) and analysis of the species specificity of stimulation by CSF-1 and Interleukin 34. Cytokine.

[36] Liu H, Leo C, Chen X, Wong BR, Williams LT, Lin H, He X (2012). The mechanism of shared but distinct CSF-1R signaling by the non-homologous cytokines IL-34 and CSF-1. Biochim Biophys Acta.

[37] Ma X, Lin WY, Chen Y, Stawicki S, Mukhyala K, Wu Y (2012). Structural basis for the dual recognition of helical cytokines IL-34 and CSF-1 by CSF-1R. Structure.

[38] Chihara T, Suzu S, Hassan R, Chutiwitoonchai N, Hiyoshi M, Motoyoshi K (2010). IL-34 and M-CSF share the receptor Fms but are not identical in biological activity and signal activation. Cell Death Differ.

[39] Eda H, Shimada H, Beidler DR, Monahan JB (2011). Proinflammatory cytokines, IL-1β and TNF-α, induce expression of interleukin-34 mRNA via JNK- and p44/42 MAPK-NF-κB pathway but not p38 pathway in osteoblasts. Rheumatol Int.

[40] Baud'Huin M, Renault R, Charrier C, Riet A, Moreau A, Brion R (2010). Interleukin-34 is expressed by giant cell tumours of bone and plays a key role in RANKL-induced osteoclastogenesis. J Pathol.

[41] Chen T, Wang X, Guo L, Wu M, Duan Z, Lv J (2014). Embryonic stem cells promoting macrophage survival and function are crucial for teratoma development. Front Immunol.

[42] Wei S, Nandi S, Chitu V, Yeung YG, Yu W, Huang M (2010). Functional overlap but differential expression of CSF-1 and IL-34 in their CSF-1 receptor-mediated regulation of myeloid cells. J Leukoc Biol.

[43] Yu Y, Yang D, Qiu L, Okamura H, Guo J, Haneji T (2014). Tumor necrosis factor-α induces interleukin-34 expression through nuclear factor-κB activation in MC3T3-E1 osteoblastic cells. Mol Med Rep.

[44] Lin K, Ma J, Peng Y, Sun M, Xu K, Wu R, Lin J (2019). Autocrine Production of Interleukin-34 Promotes the Development of Endometriosis through CSF1R/JAK3/STAT6 signaling. Sci Rep.

[45] Zhou J, Sun X, Zhang J, Yang Y, Chen D, Cao J (2018). IL-34 regulates IL-6 and IL-8 production in human lung fibroblasts via MAPK, PI3K-Akt, JAK and NF-κB signaling pathways. Int Immunopharmacol.

[46] Zins K, Heller G, Mayerhofer M, Schreiber M, Abraham D (2018). Differential prognostic impact of interleukin-34 mRNA expression and infiltrating immune cell composition in intrinsic breast cancer subtypes. Oncotarget.

[47] Truong AD, Hong Y, Lee J, Lee K, Kil DY, Lillehoj HS, Hong YH (2018). Interleukin-34 regulates Th1 and Th17 cytokine production by activating multiple signaling pathways through CSF-1R in chicken cell lines. Int J Mol Sci.

[48] Ségaliny AI, Brion R, Brulin B, Maillasson M, Charrier C, Téletchéa S, Heymann D (2015). IL-34 and M-CSF form a novel heteromeric cytokine and regulate the M-CSF receptor activation and localization. Cytokine.

[49] Detry S, Składanowska K, Vuylsteke M, Savvides SN, Bloch Y (2019). Revisiting the combinatorial potential of cytokine subunits in the IL-12 family. Biochem Pharmacol.

[50] Gorczynski RM (2020). IL-17 Signaling in the Tumor Microenvironment. Adv Exp Med Biol.

[51] Droin N, Solary E (2010). Editorial: CSF1R, CSF-1, and IL-34, a “ménage à trois” conserved across vertebrates. J Leukoc Biol.

[52] Nandi S, Cioce M, Yeung YG, Nieves E, Tesfa L, Lin H (2013). Receptor-type protein-tyrosine phosphatase ζ is a functional receptor for interleukin-34. J Biol Chem.

[53] Segaliny AI, Brion R, Mortier E, Maillasson M, Cherel M, Jacques Y (2015). Syndecan-1 regulates the biological activities of interleukin-34. Biochim Biophys Acta.

[54] Nandi S, Gokhan S, Dai XM, Wei S, Enikolopov G, Lin H (2012). The CSF-1 receptor ligands IL-34 and CSF-1 exhibit distinct developmental brain expression patterns and regulate neural progenitor cell maintenance and maturation. Dev Biol.

[55] Zwicker S, Bureik D, Bosma M, Martinez GL, Almer S, Boström EA (2016). Receptor-type protein-tyrosine phosphatase ζ and colony stimulating factor-1 receptor in the intestine: Cellular expression and cytokine- and chemokine responses by interleukin-34 and colony stimulating factor-1. PLoS ONE.

[56] Franzè E, Dinallo V, Rizzo A, Giovangiulio MD, Bevivino G, Stolfi C (2018). Interleukin-34 sustains pro-tumorigenic signals in colon cancer tissue. Oncotarget.

[57] Pixley FJ, Stanley ER (2004). CSF-1 regulation of the wandering macrophage: Complexity in action. Trends Cell Biol.

[58] Ogawa S, Matsuoka Y, Takada M, Matsui K, Yamane F, Kubota E (2019). Interleukin 34 (IL-34) cell-surface localization regulated by the molecular chaperone 78-kDa glucose-regulated protein facilitates the differentiation of monocytic cells. J Biol Chem.

[59] Boulakirba S, Pfeifer A, Mhaidly R, Obba S, Goulard M, Schmitt T (2018). IL-34 and CSF-1 display an equivalent macrophage differentiation ability but a different polarization potential. Sci Rep.

[60] Locati M, Mantovani A, Sica A (2013). Macrophage Activation and Polarization as an Adaptive Component of Innate Immunity. Adv Immunol.

[61] Murray PJ, Allen JE, Biswas SK, Fisher EA, Gilroy DW, Goerdt S (2014). Macrophage Activation and Polarization: Nomenclature and Experimental Guidelines. Immunity.

[62] Locati M, Curtale G, Mantovani A (2020). Diversity, Mechanisms, and Significance of Macrophage Plasticity. Annu Rev Pathol.

[63] Foucher ED, Blanchard S, Preisser L, Garo E, Ifrah N, Guardiola P (2013). IL-34 Induces the Differentiation of Human Monocytes into Immunosuppressive Macrophages. Antagonistic Effects of GM-CSF and IFNγ. PLoS ONE.

[64] Lindau R, Mehta RB, Lash GE, Papapavlou G, Boij R, Berg G (2018). Interleukin-34 is present at the fetal-maternal interface and induces immunoregulatory macrophages of a decidual phenotype *in vitro*. Hum Reprod.

[65] Booker BE, Clark RS, Pellom ST, Adunyah SE (2015). Interleukin-34 induces monocytic-like differentiation in leukemia cell lines. Int J Biochem Mol Biol.

[66] Popovic M, Yaparla A, Paquin-Proulx D, Koubourli DV, Webb R, Firmani M, Grayfer L (2019). Colony-stimulating factor-1- and interleukin-34-derived macrophages differ in their susceptibility to Mycobacterium marinum. J Leukoc Biol.

[67] Wang T, Kono T, Monte MM, Kuse H, Costa MM, Korenaga H (2013). Identification of IL-34 in teleost fish: Differential expression of rainbow trout IL-34, MCSF1 and MCSF2, ligands of the MCSF receptor. Mol Immunol.

[68] Xue Y, Jiang X, Gao J, Li X, Xu J, Wang J (2019). Functional characterisation of interleukin 34 in grass carp Ctenopharyngodon idella. Fish Shellfish Immunol.

[69] Shen HY, Zhou Y, Zhou QJ, Li MY, Chen J (2020). Mudskipper interleukin-34 modulates the functions of monocytes/macrophages via the colony-stimulating factor-1 receptor 1. Zool Res.

[70] Yu C, Zhang P, Zhang TF, Sun L (2020). IL-34 Regulates the Inflammatory Response and Anti-Bacterial Immune Defense of Japanese Flounder Paralichthys Olivaceus. Fish Shellfish Immunol.

[71] Hoang HH, Wang PC, Chen SC (2020). Interleukin 34 serves as a novel molecular adjuvant against nocardia seriolae infection in largemouth bass (Micropterus salmoides). Vaccines.

[72] Wu S, Xue R, Hassan S, Nguyen TML, Wang T, Pan H (2018). Il34-Csf1r Pathway Regulates the Migration and Colonization of Microglial Precursors. Dev Cell.

[73] Jiang Y, Chen J, Yen K, Xu J (2019). Ectopically Expressed IL-34 Can Efficiently Induce Macrophage Migration to the Liver in Zebrafish. Zebrafish.

[74] Kuil LE, Oosterhof N, Geurts SN, Van Der Linde HC, Meijering E, Van Ham TJ (2019). Reverse genetic screen reveals that Il34 facilitates yolk sac macrophage distribution and seeding of the brain. Dis Model Mech.

[75] Sattentau QJ, Stevenson M (2016). Macrophages and HIV-1: An Unhealthy Constellation. Cell Host Microbe.

[76] Paquin-Proulx D, Greenspun BC, Kitchen SM, Saraiva Raposo RA, Nixon DF, Grayfer L (2018). Human interleukin-34-derived macrophages have increased resistant to HIV-1 infection. Cytokine.

[77] Valcour V, Chalermchai T, Sailasuta N, Marovich M, Lerdlum S, Suttichom D (2012). Central Nervous System Viral Invasion and Inflammation During Acute HIV Infection. J Infect Dis.

[78] Mathews S, Branch Woods A, Katano I, Makarov E, Thomas MB, Gendelman HE (2019). Human Interleukin-34 facilitates microglia-like cell differentiation and persistent HIV-1 infection in humanized mice. Mol Neurodegener.

[79] Krammer F, Smith G, Fouchier R, Peiris M, Kedzierska K, Doherty P (2018). Influenza. Nat Rev Dis Primers.

[80] Yu G, Bing Y, Zhu S, Li W, Xia L, Li Y, Liu Z (2015). Activation of the Interleukin-34 Inflammatory Pathway in Response to Influenza A Virus Infection. Am J Med Sci.

[81] Preisser L, Miot C, Le Guillou-Guillemette E, Beaumont E, Foucher ED, Garo E (2014). IL-34 and Macrophage Colony-Stimulating Factor Are Overexpressed in Hepatitis C Virus Fibrosis and Induce Profibrotic Macrophages That Promote Collagen Synthesis by Hepatic Stellate Cells. Hepatology.

[82] Cheng ST, Tang H, Ren JH, Chen X, Huang AL, Juan C (2017). Interleukin-34 inhibits hepatitis B virus replication *in vitro* and *in vivo*. PLoS ONE.

[83] Wang YQ, Cao WJ, Gao YF, Ye J, Zou GZ (2018). Serum interleukin-34 Level Can Be an Indicator of Liver Fibrosis in Patients with Chronic Hepatitis B Virus Infection. World J Gastroenterol.

[84] Kong F, Zhou K, Zhu T, Lian Q, Tao Y, Li N (2019). Interleukin-34 mediated by hepatitis B virus X protein via CCAAT/enhancer-binding protein α contributes to the proliferation and migration of hepatoma cells. Cell Prolif.

[85] Tang K, Zhang C, Zhang Y, Zhang Y, Du H, Jin B, Ma Y (2019). Elevated plasma interleukin 34 levels correlate with disease severity-reflecting parameters of patients with haemorrhagic fever with renal syndrome. Infect Dis (Lond).

[86] Grayfer L, Robert J (2016). Amphibian macrophage development and antiviral defenses. Dev Comp Immunol.

[87] Yaparla A, Popovic M, Grayfer L (2018). Differentiation-dependent antiviral capacities of amphibian (Xenopus laevis) macrophages. J Biol Chem.

[88] Yaparla A, Docter-Loeb H, Melnyk MLS, Batheja A, Grayfer L (2019). The amphibian (Xenopus laevis) colony-stimulating factor-1 and interleukin-34-derived macrophages possess disparate pathogen recognition capacities. Dev Comp Immunol.

[89] Mantovani A, Marchesi F, Malesci A, Laghi L, Allavena P (2017). Tumour-associated macrophages as treatment targets in oncology. Nat Rev Clin Oncol.

[90] Williams CB, Yeh ES, Soloff AC (2016). Tumor-associated macrophages: Unwitting accomplices in breast cancer malignancy. NPJ Breast Cancer.

[91] Wang J, Li D, Cang H, Guo B (2019). Crosstalk between cancer and immune cells: Role of tumor-associated macrophages in the tumor microenvironment. Cancer Med.

[92] Kumar S, Ramesh A, Kulkarni A (2020). Targeting macrophages: a novel avenue for cancer drug discovery. Expert Opin Drug Discov.

[93] Zhou J, Tang Z, Gao S, Li C, Feng Y, Zhou X (2020). Tumor-Associated Macrophages: Recent Insights and Therapies. Front Oncol.

[94] Jeannin P, Paolini L, Adam C, Delneste Y (2018). The roles of CSFs on the functional polarization of tumor-associated macrophages. FEBS J.

[95] Ségaliny AI, Mohamadi A, Dizier B, Lokajczyk A, Brion R, Lanel R (2015). Interleukin-34 promotes tumor progression and metastatic process in osteosarcoma through induction of angiogenesis and macrophage recruitment. Int J Cancer.

[96] Baghdadi M, Wada H, Nakanishi S, Abe H, Han N, Wira EP (2016). Chemotherapy-induced IL34 enhances immunosuppression by tumor-associated macrophages and mediates survival of chemoresistant lung cancer cells. Cancer Res.

[97] Arora H, Panara K, Kuchakulla M, Kulandavelu S, Burnstein KL, Schally AV (2018). Alterations of tumor microenvironment by nitric oxide impedes castration-resistant prostate cancer growth. Proc Natl Acad Sci U S A.

[98] Kobayashi T, Baghdadi M, Han N, Murata T, Hama N, Otsuka R (2019). Prognostic value of IL-34 in colorectal cancer patients. Immunol Med.

[99] Endo H, Hama N, Baghdadi M, Ishikawa K, Otsuka R, Wada H (2019). Interleukin-34 expression in ovarian cancer: A possible correlation with disease progression. Int Immunol.

[100] Zhou SL, Hu ZQ, Zhou ZJ, Dai Z, Wang Z, Cao Y (2016). miR-28-5p-IL-34-macrophage Feedback Loop Modulates Hepatocellular Carcinoma Metastasis. Hepatology.

[101] Noda Y, Kawaguchi T, Korenaga M, Yoshio S, Komukai S, Nakano M (2019). High serum interleukin-34 level is a predictor of poor prognosis in patients with non-viral hepatocellular carcinoma. Hepatol Res.

[102] van de Laar L, Saelens W, De Prijck S, Martens L, Scott CL, Van Isterdael G (2016). Yolk Sac Macrophages, Fetal Liver, and Adult Monocytes Can Colonize an Empty Niche and Develop into Functional Tissue-Resident Macrophages. Immunity.

[103] Guilliams M, Thierry GR, Bonnardel J, Bajenoff M (2020). Establishment and Maintenance of the Macrophage Niche. Immunity.

[104] Liu W, Zhang X (2015). Receptor activator of nuclear factor-κB ligand (RANKL)/RANK/osteoprotegerin system in bone and other tissues. Mol Med Rep.

[105] Ono T, Nakashima T (2018). Recent advances in osteoclast biology. Histochem Cell Biol.

[106] Heymann D, Guicheux J, Gouin F, Passuti N, Daculsi G (1998). Cytokines, Growth Factors and Osteoclasts. Cytokine.

[107] Heymann D, Rousselle AV (2000). gp130 cytokine family and bone cells. Cytokine.

[108] Yamashita T, Takahashi N, Udagawa N (2012). New roles of osteoblasts involved in osteoclast differentiation. World J Orthop.

[109] Chen Z, Buki K, Vääräniemi J, Gu G, Väänänen HK (2011). The critical role of IL-34 in osteoclastogenesis. PLoS ONE.

[110] Cheng X, Wan QL, Li ZB (2017). AG490 suppresses interleukin-34-mediated osteoclastogenesis in mice bone marrow macrophages. Cell Biol Int.

[111] Chemel M, Le Goff B, Brion R, Cozic C, Berreur M, Amiaud J (2012). Interleukin 34 expression is associated with synovitis severity in rheumatoid arthritis patients. Ann Rheum Dis.

[112] Galligan CL, Fish EN (2017). Interleukin-34 Promotes Fibrocyte Proliferation. J Interferon Cytokine Res.

[113] Wang B, Tang Y, Sun X, Ouyang X, Li H, Wei J (2018). Increased IL-6 expression on THP-1 by IL-34 stimulation up-regulated rheumatoid arthritis Th17 cells. Clin Rheumatol.

[114] Elkhider A, Wei J, Al-Azab M, Tang Y, Walana W, Li W (2020). IL-34 Modulates Rheumatoid Synovial Fibroblasts Proliferation and Migration via ERK/AKT Signalling Pathway. Clin Exp Rheumatol.

[115] Chemel M, Brion R, Segaliny AI, Lamora A, Charrier C, Brulin B (2017). Bone Morphogenetic Protein 2 and Transforming Growth Factor β1 Inhibit the Expression of the Proinflammatory Cytokine IL-34 in Rheumatoid Arthritis Synovial Fibroblasts. Am J Pathol.

[116] Wang B, Ma Z, Wang M, Sun X, Tang Y, Li M (2017). IL-34 upregulated Th17 production through increased IL-6 expression by rheumatoid fibroblast-like synoviocytes. Mediators Inflamm.

[117] Cui My, Li X, Lei Ym, Xia Lp, Lu J, Shen H (2019). Effects of IL-34 on the secretion of RANKL/OPG by fibroblast-like synoviocytes and peripheral blood mononuclear cells in rheumatoid arthritis. Eur Cytokine Netw.

[118] Li N, Jiang L, Cai Y, Liu JY, Zhao T, Kong N (2020). The correlation between interleukin-34 and bone erosion under ultrasound in rheumatoid arthritis. Mod Rheumatol.

[119] Franzé E, Monteleone I, Cupi ML, Mancia P, Caprioli F, Marafini I (2015). Interleukin-34 sustains inflammatory pathways in the gut. Clin Sci (Lond).

[120] Zwicker S, Martinez GL, Bosma M, Gerling M, Clark R, Majster M (2015). Interleukin 34: A new modulator of human and experimental inflammatory bowel disease. Clin Sci (Lond).

[121] Franzè E, Marafini I, De Simone V, Monteleone I, Caprioli F, Colantoni A (2016). Interleukin-34 induces Cc-chemokine ligand 20 in gut epithelial cells. J Crohns Colitis.

[122] Franzè E, Dinallo V, Laudisi F, Di Grazia A, Di Fusco D, Colantoni A (2020). Interleukin-34 Stimulates Gut Fibroblasts to Produce Collagen Synthesis. J Crohns Colitis.

[123] Mizuno T (2011). Disruption of Interactions Between Immunocytes, Glia and Neurons in Demyelinating Diseases: A View From Neuroscience. Rinsho Shinkeigaku.

[124] Jin S, Sonobe Y, Kawanokuchi J, Horiuchi H, Cheng Y, Wang Y (2014). Interleukin-34 restores blood-brain barrier integrity by upregulating tight junction proteins in endothelial cells. PLoS ONE.

[125] Abdel-Dayem M, Shaker M, Gameil N (2019). Impact of interferon β-1b, interferon β-1a and fingolimod therapies on serum interleukins-22, 32α and 34 concentrations in patients with relapsing-remitting multiple sclerosis. J Neuroimmunol.

[126] Wlodarczyk A, Benmamar-Badel A, Cédile O, Jensen KN, Kramer I, Elsborg NB (2019). CSF1R stimulation promotes increased neuroprotection by CD11c+ microglia in EAE. Front Cell Neurosci.

[127] Safari-Alighiarloo N, Taghizadeh M, Mohammad Tabatabaei S, Namaki S, Rezaei-Tavirani M (2020). Identification of common key genes and pathways between type 1 diabetes and multiple sclerosis using transcriptome and interactome analysis. Endocrine.

[128] Lee PW, Selhorst A, Lampe SG, Liu Y, Yang Y, Lovett-Racke AE (2020). Neuron-Specific Vitamin D Signaling Attenuates Microglia Activation and CNS Autoimmunity. Front Neurol.

[129] Li J, Liu L, Rui W, Li X, Xuan D, Zheng S (2017). New Interleukins in Psoriasis and Psoriatic Arthritis Patients: The Possible Roles of Interleukin-33 to Interleukin-38 in Disease Activities and Bone Erosions. Dermatology.

[130] Hwang SJ, Choi B, Kang SS, Chang JH, Kim YG, Chung YH (2012). Interleukin-34 produced by human fibroblast-like synovial cells in rheumatoid arthritis supports osteoclastogenesis. Arthritis Res Ther.

[131] Tian Y, Shen H, Xia L, Lu J (2013). Elevated serum and synovial fluid levels of interleukin-34 in rheumatoid arthritis: Possible association with disease progression via interleukin-17 production. J Interferon Cytokine Res.

[132] Moon SJ, Hong YS, Ju JH, Kwok SK, Park SH, Min JK (2013). Increased Levels of Interleukin 34 in Serum and Synovial Fluid Are Associated With Rheumatoid Factor and Anticyclic Citrullinated Peptide Antibody Titers in Patients With Rheumatoid Arthritis. J Rheumatol.

[133] Chang SH, Choi BY, Choi J, Yoo JJ, Ha YJ, Cho HJ (2015). Baseline serum interleukin-34 levels independently predict radiographic progression in patients with rheumatoid arthritis. Rheumatol Int.

[134] Zhang F, Ding R, Li P, Ma C, Song D, Wang X (2015). Interleukin-34 in Rheumatoid Arthritis: Potential Role in Clinical Therapy. Int J Clin Exp Med.

[135] Ding R, Li P, Song D, Zhang X, Bi L (2015). Predictors of response to TNF-α antagonist therapy in Chinese rheumatoid arthritis. Clin Rheumatol.

[136] Garcia S, Hartkamp LM, Malvar-Fernandez B, van Es IE, Lin H, Wong J (2016). Colony-stimulating factor (CSF) 1 receptor blockade reduces inflammation in human and murine models of rheumatoid arthritis. Arthritis Res Ther.

[137] Yang S, Jiang S, Wang Y, Tu S, Wang Z, Chen Z (2016). Interleukin 34 upregulation contributes to the increment of MicroRNA 21 expression through STAT3 activation associated with disease activity in rheumatoid arthritis. J Rheumatol.

[138] Ding LL, Li X, Lei YM, Xia LP, Lu J, Shen H (2020). Effect of Interleukin-34 on Secretion of Angiogenesis Cytokines by Peripheral Blood Mononuclear Cells of Rheumatoid Arthritis. Immunol Invest.

[139] Ciccia F, Alessandro R, Rodolico V, Guggino G, Raimondo S, Guarnotta C (2013). IL-34 is overexpressed in the inflamed salivary glands of patients with Sjögren's syndrome and is associated with the local expansion of pro-inflammatory CD14brightCD16+ monocytes. Rheumatology (Oxford).

[140] Liu Y, Zhang B, Lei Y, Xia L, Lu J, Shen H (2020). Serum Levels of interleukin-34 and Clinical Correlation in Patients With Primary Sjögren's Syndrome. Int J Rheum Dis.

[141] Wang H, Cao J, Lai X (2017). Serum interleukin-34 levels are elevated in patients with systemic lupus erythematosus. Molecules.

[142] Xie HH, Shen H, Zhang L, Cui MY, Xia LP, Lu J (2018). Elevated Serum Interleukin-34 Level in Patients with Systemic Lupus Erythematosus Is Associated with Disease Activity. Sci Rep.

[143] Wada Y, Gonzalez-Sanchez HM, Weinmann-Menke J, Iwata Y, Ajay AK, Meineck M (2019). IL-34-Dependent intrarenal and systemic mechanisms promote lupus nephritis in MRL-Faslpr mice. J Am Soc Nephrol.

[144] El-Banna HS, El Khouly RM, Gado SE (2020). Elevated Serum interleukin-34 Level in Juvenile Systemic Lupus Erythematosus and Disease Activity. Clin Rheumatol.

[145] Abdel-Rehim ASM, Mohamed NA, Shakweer MM (2020). Interleukin-34 as a marker for subclinical proliferative lupus nephritis. Lupus.

[146] Kuzumi A, Yoshizaki A, Toyama S, Fukasawa T, Ebata S, Nakamura K (2018). Serum interleukin-34 levels in patients with systemic sclerosis: Clinical association with interstitial lung disease. J Dermatol.

[147] Rolla G, Fusaro E, Nicola S, Bucca C, Peroni C, Parisi S (2016). Th-17 cytokines and interstitial lung involvement in systemic sclerosis. J Breath Res.

[148] Boström EA, Lundberg P (2013). The newly discovered cytokine IL-34 is expressed in gingival fibroblasts, shows enhanced expression by pro-inflammatory cytokines, and stimulates osteoclast differentiation. PLoS ONE.

[149] Kawabe M, Ohyama H, Kato-Kogoe N, Yamada N, Yamanegi K, Nishiura H (2015). Expression of interleukin-34 and colony stimulating factor-1 in the stimulated periodontal ligament cells with tumor necrosis factor-α. Med Mol Morphol.

[150] Batra P, Das S, Patel P (2019). Comparative evaluation of Gingival Crevicular Fluid (GCF) levels of Interleukin-34 levels in periodontally healthy and in patients with chronic and aggressive periodontitis- A cross-sectional study. Saudi Dent J.

[151] Baghdadi M, Ishikawa K, Nakanishi S, Murata T, Umeyama Y, Kobayashi T (2019). A role for IL-34 in osteolytic disease of multiple myeloma. Blood Adv.

[152] Nakajima K, Kohsaka S (2001). Microglia: Activation and Their Significance in the Central Nervous System. J Biochem.

[153] Cahoy JD, Emery B, Kaushal A, Foo LC, Zamanian JL, Christopherson KS (2008). A transcriptome database for astrocytes, neurons, and oligodendrocytes: A new resource for understanding brain development and function. J Neurosci.

[154] Mizuno T, Doi Y, Mizoguchi H, Jin S, Noda M, Sonobe Y (2011). Interleukin-34 selectively enhances the neuroprotective effects of microglia to attenuate oligomeric amyloid-β neurotoxicity. Am J Pathol.

[155] Easley-Neal C, Foreman O, Sharma N, Zarrin AA, Weimer RM (2019). CSF1R Ligands IL-34 and CSF1 Are Differentially Required for Microglia Development and Maintenance in White and Gray Matter Brain Regions. Front Immunol.

[156] O'Koren EG, Yu C, Klingeborn M, Wong AYW, Prigge CL, Mathew R (2019). Microglial Function Is Distinct in Different Anatomical Locations during Retinal Homeostasis and Degeneration. Immunity.

[157] Kaucká M, Adameyko I (2014). Non-canonical Functions of the Peripheral Nerve. Exp Cell Res.

[158] Zigmond RE, Echevarria FD (2019). Macrophage Biology in the Peripheral Nervous System After Injury. Prog Neurobiol.

[159] Wang PL, Yim AKY, Kim KW, Avey D, Czepielewski RS, Colonna M (2020). Peripheral Nerve Resident Macrophages Share Tissue-Specific Programming and Features of Activated Microglia. Nat Commun.

[160] Wright-Jin EC, Gutmann DH (2019). Microglia as Dynamic Cellular Mediators of Brain Function. Trends Mol Med.

[161] Ma D, Doi Y, Jin S, Li E, Sonobe Y, Takeuchi H (2012). TGF-β induced by interleukin-34-stimulated microglia regulates microglial proliferation and attenuates oligomeric amyloid β neurotoxicity. Neurosci Lett.

[162] Wlodarczyk A, Holtman IR, Krueger M, Yogev N, Bruttger J, Khorooshi R (2017). A novel microglial subset plays a key role in myelinogenesis in developing brain. EMBO J.

[163] Walker DG, Tang TM, Lue LF (2017). Studies on colony stimulating factor receptor-1 and ligands colony stimulating factor-1 and interleukin-34 in Alzheimer's disease brains and human microglia. Front Aging Neurosci.

[164] Khoshnan A, Sabbaugh A, Calamini B, Marinero SA, Dunn DE, Yoo JH (2017). IKK β and mutant huntingtin interactions regulate the expression of IL-34: Implications for microglialmediated neurodegeneration in HD. Hum Mol Genet.

[165] Lemus HN, Warrington AE, Rodriguez M (2018). Multiple Sclerosis: Mechanisms of Disease and Strategies for Myelin and Axonal Repair. Neurol Clin.

[166] Vasek MJ, Garber C, Dorsey D, Durrant DM, Bollman B, Soung A (2016). A complement-microglial axis drives synapse loss during virus-induced memory impairment. Nature.

[167] Zhu C, Herrmann US, Falsig J, Abakumova I, Nuvolone M, Schwarz P (2016). A neuroprotective role for microglia in prion diseases. J Exp Med.

[168] Hoeffel G, Wang Y, Greter M, See P, Teo P, Malleret B (2012). Adult Langerhans cells derive predominantly from embryonic fetal liver monocytes with a minor contribution of yolk sac-derived macrophages. J Exp Med.

[169] Wang Y, Bugatti M, Ulland TK, Vermi W, Gilfillan S, Colonna M (2016). Nonredundant roles of keratinocyte-derived IL-34 and neutrophil-derived CSF1 in Langerhans cell renewal in the steady state and during inflammation. Eur J Immunol.

[170] Lonardi S, Scutera S, Licini S, Lorenzi L, Cesinaro AM, Benerini Gatta L (2020). CSF1R is required for differentiation and migration of Langerhans cells and Langerhans cell histiocytosis. Cancer Immunol Res.

[171] Han N, Baghdadi M, Ishikawa K, Endo H, Kobayashi T, Wada H (2018). Enhanced IL-34 expression in Nivolumab-resistant metastatic melanoma. Inflamm Regen.

[172] Giricz O, Mo Y, Dahlman KB, Cotto-Rios XM, Vardabasso C, Nguyen H (2018). The RUNX1/IL-34/CSF-1R axis is an autocrinally regulated modulator of resistance to BRAF-V600E inhibition in melanoma. JCI Insight.

[173] Platt AM, Bain CC, Bordon Y, Sester DP, Mowat AM (2010). An Independent Subset of TLR Expressing CCR2-Dependent Macrophages Promotes Colonic Inflammation. J Immunol.

[174] Geissmann F, Manz MG, Jung S, Sieweke MH, Merad M, Ley K (2010). Development of Monocytes, Macrophages, and Dendritic Cells. Science.

[175] Guilliams M, Mildner A, Yona S (2018). Developmental and Functional Heterogeneity of Monocytes. Immunity.

[176] Bain CC, Scott CL, Uronen-Hansson H, Gudjonsson S, Jansson O, Grip O (2013). Resident and pro-inflammatory macrophages in the colon represent alternative context-dependent fates of the same Ly6C hi monocyte precursors. Mucosal Immunol.

[177] Li P, He K, Li J, Liu Z, Gong J (2017). The role of Kupffer cells in hepatic diseases. Mol Immunol.

[178] Atif M, Conti F, Gorochov G, Oo YH, Miyara M (2020). Regulatory T cells in solid organ transplantation. Clin Transl Immunology.

[179] Bézie S, Picarda E, Ossart J, Tesson L, Usal C, Renaudin K (2015). IL-34 is a Treg-specific cytokine and mediates transplant tolerance. J Clin Invest.

[180] Baek JH, Zeng R, Weinmann-Menke J, Valerius MT, Wada Y, Ajay AK (2015). IL-34 mediates acute kidney injury and worsens subsequent chronic kidney disease. J Clin Invest.

[181] Shoji H, Yoshio S, Mano Y, Kumagai E, Sugiyama M, Korenaga M (2016). Interleukin-34 as a fibroblast-derived marker of liver fibrosis in patients with non-alcoholic fatty liver disease. Scientific Reports.

[182] Chen L, Yu Y, Liu E, Duan L, Zhu D, Chen J (2019). Schistosoma japonicum soluble egg antigen inhibits TNF-α-induced IL-34 expression in hepatic stellate cells. Parasitol Res.

[183] Weissleder R, Nahrendorf M, Pittet MJ (2014). Imaging Macrophages with Nanoparticles. Nat Mater.

[184] Saeed M, Gao J, Shi Y, Lammers T, Yu H (2019). Engineering Nanoparticles to Reprogram the Tumor Immune Microenvironment for Improved Cancer Immunotherapy. Theranostics.

[185] Shubayev VI, Pisanic 2nd TR, Jin S (2009). Magnetic Nanoparticles for Theragnostics. Adv Drug Deliv Rev.

[186] Caspani S, Magalhães R, Araújo JP, Tavares Sousa C (2020). Magnetic Nanomaterials as Contrast Agents for MRI. Materials (Basel).

[187] Dadfar SM, Roemhild K, Drude NI, von Stillfried S, Knüchel R, Kiessling F (2019). Iron Oxide Nanoparticles: Diagnostic, Therapeutic and Theranostic Applications. Adv Drug Deliv Rev.

[188] Bouvain P, Temme S, Flögel U (2020). Hot Spot 19 F Magnetic Resonance Imaging of Inflammation. Wiley Interdiscip Rev Nanomed Nanobiotechnol.

[189] Vaz SC, Oliveira F, Herrmann K, Veit-Haibach P (2020). Nuclear Medicine and Molecular Imaging Advances in the 21st Century. Br J Radiol.

[190] Lee ST, Burvenich I, Scott AM (2019). Novel Target Selection for Nuclear Medicine Studies. Semin Nucl Med.

[191] Mukherjee S, Sonanini D, Maurer A, Daldrup-Link HE (2019). The Yin and Yang of Imaging Tumor Associated Macrophages With PET and MRI. Theranostics.

[192] Burke BP, Cawthorne C, Archibald SJ (2017). Multimodal Nanoparticle Imaging Agents: Design and Applications. Philos Trans A Math Phys Eng Sci.

[193] Talbot A, Devos L, Dubus F, Vermandel M (2020). Multimodal Imaging in Radiotherapy: Focus on Adaptive Therapy and Quality Control. Cancer Radiother.

[194] Zhang C, Yu X, Gao L, Zhao Y, Lai J, Lu D (2017). Noninvasive Imaging of CD206-Positive M2 Macrophages as an Early Biomarker for Post-Chemotherapy Tumor Relapse and Lymph Node Metastasis. Theranostics.

[195] Zang X, Zhang X, Hu H, Qiao M, Zhao X, Deng Y (2019). Targeted Delivery of Zoledronate to Tumor-Associated Macrophages for Cancer Immunotherapy. Mol. Pharmaceutics.

[196] Han J, Zhen J, Nguyen VD, Go G, Choi Y, Ko SY, Park JPS (2016). Hybrid-Actuating Macrophage-Based Microrobots for Active Cancer Therapy. Sci Rep.

[197] Xie Z, Su Y, Kim GB, Selvi E, Ma C, Aragon-Sanabria V (2017). Immune Cell-Mediated Biodegradable Theranostic Nanoparticles for Melanoma Targeting and Drug Delivery. Small.

[198] Evans MA, Huang PJ, Iwamoto Y, Ibsen KN, Chan EM, Hitomi Y (2018). Macrophage-mediated Delivery of Light Activated Nitric Oxide Prodrugs with Spatial, Temporal and Concentration Control. Chem Sci.

[199] Zheng B, Bai Y, Chen H, Pan H, Ji W, Gong X (2018). Targeted Delivery of Tungsten Oxide Nanoparticles for Multifunctional Anti-Tumor Therapy via Macrophages. Biomater Sci.

[200] Suk JS, Xu Q, Kim N, Hanes J, Ensign LM (2016). PEGylation as a Strategy for Improving Nanoparticle-Based Drug and Gene Delivery. Adv Drug Deliv Rev.

[201] Paolino D, Accolla ML, Cilurzo F, Cristiano MC, Cosco D, Castelli F (2017). Interaction Between PEG Lipid and DSPE/DSPC Phospholipids: An Insight of PEGylation Degree and Kinetics of de-PEGylation. Colloids Surf B Biointerfaces.

[202] Zhu X, Tao W, Liu D, Wu J, Guo Z, Ji X (2017). Surface De-PEGylation Controls Nanoparticle-Mediated siRNA Delivery *in vitro* and *in vivo*. Theranostics.

[203] D'Acunto M (2018). Detection of Intracellular Gold Nanoparticles: An Overview. Materials (Basel).

[204] An L, Wang Y, Lin J, Tian Q, Xie Y, Hu J (2019). Macrophages-Mediated Delivery of Small Gold Nanorods for Tumor Hypoxia Photoacoustic Imaging and Enhanced Photothermal Therapy. ACS Appl Mater Interfaces.

[205] Nguyen VD, Min HK, Kim DH, Kim CS, Han J, Park JO (2020). Macrophage-Mediated Delivery of Multifunctional Nanotherapeutics for Synergistic Chemo-Photothermal Therapy of Solid Tumors. ACS Appl Mater Interfaces.

[206] Park JH, Cho HJ, Yoon HY, Yoon IS, Ko SH, Shim JS (2014). Hyaluronic Acid Derivative-coated Nanohybrid Liposomes for Cancer Imaging and Drug Delivery. J. Controlled Release.

[207] Guo L, Zhang Y, Wei R, Wang C, Min Feng M (2019). Lipopolysaccharide-anchored Macrophages Hijack Tumor Microtube Networks for Selective Drug Transport and Augmentation of Antitumor Effects in Orthotopic Lung Cancer. Theranostics.

[208] Meng QF, Rao L, Zan M, Chen M, Yu GT, Wei X (2018). Macrophage Membrane-Coated Iron Oxide Nanoparticles for Enhanced Photothermal Tumor Therapy. Nanotechnology.

[209] Narain A, Asawa S, V C, Patil-Sen Y (2017). Cell membrane coated nanoparticles: Next-generation therapeutics. Nanomedicine (Lond).

[210] Xu Q, Wan J, Bie N, Song X, Yang X, Yong Y (2018). A Biomimetic Gold Nanocages-Based Nanoplatform for Efficient Tumor Ablation and Reduced Inflammation. Theranostics.

[211] Muthana M, Giannoudis A, Scott SD, Fang HY, Coffelt SB, Morrow FJ (2011). Use of Macrophages to Target Therapeutic Adenovirus to Human Prostate Tumors. Cancer Res.

[212] Cheng WS, Dzojic H, Nilsson B, Tötterman TH, Essand M (2006). An Oncolytic Conditionally Replicating Adenovirus for Hormone-Dependent and Hormone-Independent Prostate Cancer. Cancer Gene Ther.

[213] Muthana M, Rodrigues S, Chen YY, Welford A, Hughes R, Tazzyman S (2013). Macrophage Delivery of an Oncolytic Virus Abolishes Tumor Regrowth and Metastasis After Chemotherapy or Irradiation. Cancer Res.

[214] Boemo MA, Byrne HM (2019). Mathematical Modelling of a Hypoxia-Regulated Oncolytic Virus Delivered by Tumour-Associated Macrophages. J Theor Biol.

[215] Din MI, Rafique F, Hussain MS, Mehmood HA, Waseem S (2019). Recent Developments in the Synthesis and Stability of Metal Ferrite Nanoparticles. Sci Prog.

[216] Yang SH, Heo D, Park J, Na S, Suh JS, Haam S (2012). Role of Surface Charge in Cytotoxicity of Charged Manganese Ferrite Nanoparticles Towards Macrophages. Nanotechnology.

[217] Jiang P, Gao W, Ma T, Wang R, Piao Y, Dong X (2019). CD137 Promotes Bone Metastasis of Breast Cancer by Enhancing the Migration and Osteoclast Differentiation of monocytes/macrophages. Theranostics.

[218] Dougherty TJ, Gomer CJ, Henderson BW, Jori G, Kessel D, Korbelik M (1998). Photodynamic Therapy. J Natl Cancer Inst.

[219] Ben-Nun Y, Merquiol E, Brandis A, Turk B, Scherz A, Blum G (2015). Photodynamic Quenched Cathepsin Activity Based Probes for Cancer Detection and Macrophage Targeted Therapy. Theranostics.

[220] Rudzińska M, Parodi A, Soond SM, Vinarov AZ, Korolev DO, Morozov AO (2019). The Role of Cysteine Cathepsins in Cancer Progression and Drug Resistance. Int J Mol Sci.

[221] Gao X, Mao D, Zuo X, Hu F, Cao J, Zhang P (2019). Specific Targeting, Imaging, and Ablation of Tumor-Associated Macrophages by Theranostic Mannose-AIEgen Conjugates. Anal Chem.

[222] Zhu L, Wei H, Wu Y, Yang S, Xiao L, Zhang J (2012). Licorice Isoliquiritigenin Suppresses RANKL-induced Osteoclastogenesis *in vitro* and prevents Inflammatory Bone Loss *in vivo*. Int J Biochem Cell Biol.

[223] Sun X, Zhang J, Wang Z, Liu B, Zhu S, Zhu L (2019). Licorice Isoliquiritigenin-Encapsulated Mesoporous Silica Nanoparticles for Osteoclast Inhibition and Bone Loss Prevention. Theranostics.

[224] Zheng JH, Nguyen VH, Jiang SN, Park SH, Tan W, Hong SH (2017). Two-step Enhanced Cancer Immunotherapy With Engineered Salmonella typhimurium Secreting Heterologous Flagellin. Sci Transl Med.

[225] Zhao J, Zhang Z, Xue Y, Wang G, Cheng Y, Pan Y (2018). Anti-tumor Macrophages Activated by Ferumoxytol Combined or Surface-Functionalized With the TLR3 Agonist Poly (I: C) Promote Melanoma Regression. Theranostics.

[226] Rodell CB, Arlauckas SP, Cuccarese MF, Garris CS, Ran Li R, Ahmed MS (2018). TLR7/8-agonist-loaded Nanoparticles Promote the Polarization of Tumour-Associated Macrophages to Enhance Cancer Immunotherapy. Nat Biomed Eng.

[227] Rodell CB, Ahmed MS, Garris CS, Pittet MJ, Weissleder R (2019). Development of Adamantane-Conjugated TLR7/8 Agonists for Supramolecular Delivery and Cancer Immunotherapy. Theranostics.

[228] Rayahin JE, Buhrman JS, Zhang Y, Koh TJ, Gemeinhart RA (2015). High and Low Molecular Weight Hyaluronic Acid Differentially Influence Macrophage Activation. ACS Biomater Sci Eng.

[229] Zhang H, Zhang X, Ren Y, Cao F, Hou L, Zhang Z (2019). An *in situ* Microenvironmental Nano-Regulator to Inhibit the Proliferation and Metastasis of 4T1 Tumor. Theranostics.

[230] Udomsinprasert W, Jittikoon J, Honsawek S (2019). Interleukin-34 as a promising clinical biomarker and therapeutic target for inflammatory arthritis. Cytokine Growth Factor Rev.

[231] Ge Y, Huang M, Yao YM (2019). Immunomodulation of interleukin-34 and its potential significance as a disease biomarker and therapeutic target. Int J Biol Sci.

[232] Cassetta L, Pollard JW (2018). Targeting macrophages: Therapeutic approaches in cancer. Nat Rev Drug Discov.

[233] Xun Q, Wang Z, Hu X, Ding K, Lu X (2020). Small-Molecule CSF1R Inhibitors as Anticancer Agents. Curr Med Chem.

[234] Kumari A, Silakari O, Singh RK (2018). Recent advances in colony stimulating factor-1 receptor/c-FMS as an emerging target for various therapeutic implications. Biomed Pharmacother.

[235] Cannarile MA, Weisser M, Jacob W, Jegg AM, Ries CH, Rüttinger D (2017). Colony-stimulating factor 1 receptor (CSF1R) inhibitors in cancer therapy. J Immunother Cancer.

[236] Xu WD, Huang AF, Fu L, Liu XY, Su LC (2019). Targeting IL-34 in inflammatory autoimmune diseases. J Cell Physiol.

[237] Yin M, Guo Y, Hu R, Cai WL, Li Y, Pei S (2020). Potent BRD4 inhibitor suppresses cancer cell-macrophage interaction. Nat Commun.

[238] Zhang J, Zu Y, Dhanasekara CS, Li J, Wu D, Fan Z (2017). Detection and Treatment of Atherosclerosis Using Nanoparticles. Wiley Interdiscip Rev Nanomed Nanobiotechnol.

[239] Guihard P, Danger Y, Brounais B, David E, Brion R, Delecrin J (2012). Induction of osteogenesis in mesenchymal stem cells by activated monocytes/macrophages depends on oncostatin M signaling. Stem Cells.

